# LC-SVC: A Low-Complexity Sparse Vector Coding Scheme for Reliable Short-Packet Transmission in MIMO-Based Wireless Sensor Systems

**DOI:** 10.3390/s26165277

**Published:** 2026-08-20

**Authors:** Xiaotong Shi, Shuyi Zhang, Junxiang Liao, Bin Zhao, Min Fang, Cheng Zeng

**Affiliations:** 1 School of Artificial Intelligence, Hubei University, Wuhan 430062, China; xiaotongshi@hubu.edu.cn (X.S.); shuyizhang@stu.hubu.edu.cn (S.Z.); 202521121013108@stu.hubu.edu.cn (J.L.); 2 Key Laboratory of Intelligent Sensing System and Security, Hubei University, Ministry of Education, Wuhan 430062, China; 3 Intelligent Sensing Center, Hubei University, Wuhan 430062, China; 4 CICT Mobile Communication Technology Co., Ltd., Wuhan 430223, China; zhaobin7@cictmobile.com (B.Z.); fangmin@cictmobile.com (M.F.)

**Keywords:** MIMO, wireless sensing gateway, short-packet transmission, sparse vector coding, binary spreading matrix, sparse recovery, orthogonal matching pursuit

## Abstract

Reliable short-packet transmission is essential for MIMO-based wireless sensor systems, where low-power terminals upload short data blocks to multi-antenna gateways under stringent latency and complexity constraints. Although multi-antenna combining improves the equivalent received signal-to-noise ratio, the post-combining recovery of sparse-vector-coded packets is still limited by the high inter-column correlation of short binary spreading matrices and redundant searches over invalid indices in conventional orthogonal matching pursuit. To address these issues, this paper proposes a low-complexity sparse vector coding scheme for reliable short-packet transmission in MIMO-based wireless sensor systems. Specifically, at the transmitter, a low-coherence binary spreading matrix (LCB-SM) construction algorithm is designed based on Hadamard initialization and column-wise correlation optimization, which improves the distinguishability of sparse support positions while preserving multiplication-free encoding. At the receiver, a frozen-index-pruned orthogonal (FIP-OMP) matching pursuit algorithm is developed to exploit the predefined sparse mapping rule, thereby excluding invalid indices during atom selection and reducing noise-induced false support detection. Simulation results show that, for N=24, $L_{s}=16$, and K=2, the LCB-SM achieves a signal-to-noise (SNR) ratio gain of approximately 1.2 dB over the optimized partial hadamard matrix (OPHM) at a BLER of $10^{-2}$; for K=3, it reduces the high-SNR BLER by more than two orders of magnitude compared with OPHM. Meanwhile, the FIP-OMP achieves an approximately 0.5–0.8 dB gain over conventional orthogonal matching pursuit at a BLER of $10^{-2}$ with an average decoding time close to that of orthogonal matching pursuit (OMP) and much lower than that of multipath matching pursuit, Dynamic OMP and compressive sampling matching pursuit. A software defined radio-based wireless experiment further validates the reconstruction capability of LC-SVC for continuous sensing data, achieving an RMSE of 0.0612. These results demonstrate that LC-SVC improves the reliability of the considered short-packet recovery task with lightweight transmitter-side encoding and low gateway-side decoding overhead.

## 1. Introduction

Reliable short-packet transmission has become a fundamental requirement in MIMO-based wireless sensor systems, where low-power sensing terminals frequently upload short data blocks to multi-antenna gateways [1,2,3,4,5,6]. These packets may contain tactile information, fingertip pressure, force-feedback measurements, contact states, or other quantized sensing data [7,8,9]. Since such sensing data are directly related to state estimation, feedback control, and real-time decision-making, packet recovery errors may cause discontinuous sensing, incorrect state judgment, or unstable control responses. Meanwhile, sensing terminals are usually constrained by battery capacity, hardware size, and computational capability [1,4,9]. Multi-antenna reception at the gateway can improve the equivalent received signal-to-noise ratio (SNR) through spatial diversity and signal combining [10,11,12,13]. However, short sensing packets contain limited redundancy and must be recovered within a short processing time [6,14,15,16,17]. Therefore, a low-complexity and reliable coding scheme is required for short-packet transmission in MIMO-based wireless sensor systems.

### 1.1. Background

In a MIMO-based wireless sensing transmission chain, a sensor node first converts physical measurements into short information packets and sends them to a multi-antenna gateway [1,10,11]. The gateway can employ maximum-ratio combining or other receive-diversity techniques to obtain an equivalent observation with an enhanced SNR. This engineering structure is attractive for wireless sensing applications because it keeps the sensor-side transmitter simple while shifting more signal processing tasks to the gateway. However, short sensing packets contain limited redundancy and must be recovered within a short processing time [6,14,15,16,17]. Therefore, the transmission scheme should not only benefit from MIMO reception but also provide lightweight encoding and fast recovery for short packets [6].

Sparse vector coding (SVC) provides a promising solution for this requirement [18,19,20,21]. In SVC, information bits are mapped to the support positions of a sparse vector, and the packet is recovered by detecting the support set at the receiver. For wireless sensing applications, the sparse support set can be interpreted as an index-domain representation of quantized sensing states, such as pressure levels, force-feedback values, or contact-state information. This representation is suitable for short packets because a small number of information bits can be embedded into sparse support positions. When a binary spreading matrix is adopted, the sensor-side codeword can be generated through sign flipping and accumulation, avoiding complex multiplication operations [22,23,24]. After multi-antenna reception and combining, the backend recovery problem can still be formulated as sparse support detection. Therefore, SVC provides a suitable coding framework for developing a short-packet transmission scheme based on MIMO wireless sensing system engineering.

### 1.2. Related Work

Existing SVC studies have improved short-packet transmission mainly from the perspectives of sparse mapping, spreading matrix design, and sparse recovery [18,20,21]. In terms of sparse mapping, pairwise grouping-based sparse mapping improves the utilization of support combinations by grouping index pairs, while sparse extended vector coding reduces the storage burden caused by high-dimensional index tables through an extended mapping structure. Block sparse vector coding introduces block-level sparsity to improve coding reliability and spectral efficiency [18]. In addition, multiple-mode sparse mapping and multi-user SVC further extend SVC to dense short-packet access scenarios such as industrial Internet of Things (IIoT) and grant-free transmission [20,21,25,26,27,28,29]. These schemes improve the flexibility or transmission efficiency of SVC, but they usually rely on more complicated mapping structures, additional index organization, or richer symbol-domain representations. For low-power wireless sensing nodes, such increased mapping and processing overhead may weaken the computational advantage of SVC [1].

The spreading matrix is another critical factor that determines the support recovery reliability of SVC. Random Bernoulli matrices are simple and widely used, but their inter-column correlation is not explicitly controlled [22,23,24]. Compressed sensing has also been applied to device discovery, algebraic reconstruction, and specialized sensing architectures, which further shows its relevance to sparse wireless sensing and communication systems [30,31,32]. In short-packet transmission, where the spreading length is limited, highly correlated columns may appear and cause confusion between different support positions. Structured binary matrices, such as Toeplitz-type or binary tree-based constructions, can reduce storage overhead and simplify hardware implementation, but they do not necessarily minimize the worst-case column correlation or guarantee column orthogonality [22,23,24]. However, when the sparse vector dimension is larger than the spreading length, the Hadamard matrix cannot provide enough orthogonal columns, and additional columns must be constructed. Some optimized matrix construction methods reduce mutual coherence through greedy row deletion or column expansion, but greedy operations may fall into local optima and may not fully suppress high-correlation column pairs under the binary constraint [23,33,34]. Therefore, for a low-complexity SVC scheme based on MIMO wireless sensing system engineering, it is necessary to design a binary spreading matrix that preserves simple sensor-side implementation while improving the distinguishability of sparse support positions.

Receiver-side sparse recovery algorithms have also been widely investigated for SVC decoding. Orthogonal matching pursuit (OMP) is attractive because of its simple greedy structure and low computational complexity [34,35,36]. However, conventional OMP performs atom selection over the full index space and is sensitive to noise-induced correlation peaks. When the spreading matrix contains correlated columns, OMP may select an incorrect atom and cause block recovery failure. Dynamic OMP is a recent OMP-type variant that adaptively updates the candidate set during greedy search, providing a useful reliability–complexity tradeoff [35]. To improve recovery reliability, compressive sampling matching pursuit (CoSaMP) introduces candidate set selection and backtracking, while multipath matching pursuit (MMP) maintains multiple search paths to reduce the risk of wrong greedy decisions [24,34,36]. These methods can improve accuracy in some cases, but they require additional residual updates, least-squares estimations, or path management operations. Such computational overhead is undesirable for real-time sensing applications where short packets must be recovered within a limited processing time [6,7,37].

In addition to conventional greedy recovery algorithms, structured or prior-informed sparse recovery methods have been developed to exploit prior information during support detection. For example, prior-support-aided recovery methods incorporate partially known support information into the reconstruction process, weighted sparse recovery assigns nonuniform weights according to the reliability of different support positions, and model-based compressed sensing restricts the feasible support set by predefined structural models such as block, tree, or group sparsity [36,38,39,40]. Structured greedy algorithms can improve recovery accuracy when the assumed prior model is accurate and consistent with the true sparse signal structure. However, these methods usually rely on externally available support priors, estimated support probabilities, or signal models that describe physical sparsity patterns. Such priors are different from the deterministic mapping-induced prior available in SVC, where the predefined bit-to-support mapping rule directly determines which support combinations are valid and which candidate indices can never appear in transmitted packets.

More importantly, most existing recovery algorithms do not fully exploit the structural redundancy of SVC mapping. In SVC, the number of all possible support combinations is usually larger than the number of valid information messages [18,20,21]. Therefore, some support combinations or index regions never correspond to valid sensing packets. Conventional OMP, CoSaMP, and MMP still search over these invalid regions during atom selection [34,35,36]. Under noise, an invalid index may generate a large matching value and be mistakenly selected into the support set, leading to packet recovery failure. This indicates that the unused index regions in SVC should not only be regarded as mapping redundancy, but can also be transformed into prior information for receiver-side pruning. Different from prior-support OMP, weighted OMP, and model-based compressed sensing, the proposed FIP-OMP does not require externally estimated support probabilities, partially known support sets, or additional block/tree/group sparsity assumptions. Instead, it exploits the deterministic invalid-index prior induced by the SVC bit-to-support mapping rule and excludes impossible candidate indices during atom selection. Therefore, the receiver-side pruning strategy in FIP-OMP is directly matched to the coding structure rather than imposed as a generic sparse-signal model.

Based on the above analysis, existing SVC schemes still have difficulty simultaneously satisfying the requirements of low sensor-side encoding complexity, low inter-column correlation, low receiver-side recovery overhead, and mapping-aware detection. Random or structured binary spreading matrices are attractive for simple implementation, but they do not explicitly address the worst-case support ambiguity caused by highly correlated columns under short spreading lengths. Meanwhile, conventional and prior-informed OMP-type recovery algorithms can exploit different forms of sparse recovery information, but they do not specifically convert the unused support regions introduced by the finite SVC mapping rule into a deterministic frozen-index-pruning mechanism. Therefore, a joint transmitter–receiver design is needed, in which the transmitter improves the geometric distinguishability of sparse support positions and the receiver avoids redundant searches over invalid candidate indices.

### 1.3. Contributions

Therefore, this work develops a low-complexity sparse vector coding scheme (LC-SVC) from the perspective of a MIMO wireless sensing system engineering chain. Unlike schemes that improve reliability by increasing mapping complexity, introducing richer symbol-domain representations, or expanding the receiver-side search tree, LC-SVC improves short-packet recovery reliability through efficient transmitter-side representation and mapping-aware receiver-side detection. The novelty of LC-SVC lies in combining a transmitter-side low-coherence binary matrix design with a receiver-side mapping-aware greedy recovery strategy. Specifically, the proposed LCB-SM explicitly suppresses high-correlation column pairs under the binary spreading constraint, while the proposed FIP-OMP exploits the unused support structure of SVC to exclude invalid candidates during atom selection. In this way, matrix-induced support ambiguity and redundant full-index searches are addressed simultaneously.

The main contributions of this paper are summarized as follows:

1. An LC-SVC framework is proposed for reliable MIMO-based wireless sensing short-packet transmission. The proposed framework maps sensing short packets into sparse support patterns and recovers them after multi-antenna combining at the gateway. This design is motivated by practical wireless sensing scenarios in which the sensor node has limited computational capability, while the gateway can afford more signal processing.
2. A transmitter-side LCB-SM is designed for reliable short-packet sparse encoding. The proposed method constructs orthogonal initial columns using a Hadamard matrix and then reduces the maximum correlation between each newly added column and the existing columns under the binary constraint. This design reduces support ambiguity caused by highly correlated short spreading columns. The matrix can be generated offline and stored at both the transmitter and receiver. During online sensor-side encoding, only addition and subtraction operations are required.
3. A receiver-side frozen-index-pruned orthogonal matching pursuit (FIP-OMP) algorithm is developed for mapping-aware and efficient sparse support recovery. Different from conventional OMP-type decoders that search over the full index space, the algorithm establishes a frozen set according to the indices that are not used in the predefined mapping rule and excludes these indices during the atom selection stage. Therefore, noise-induced erroneous selection can be reduced while maintaining the same order of complexity as conventional OMP.
4. An SDR-based wireless sensing testbed is constructed using a USRP-LW B210 platform and a SEED Robotics RH8D (Seed Robotics, Lisbon, Portugal) dexterous-hand sensing device. The measured results verify the capability of LC-SVC to reconstruct continuous fingertip force-sensing data over a real wireless channel, demonstrating its practical feasibility in MIMO-based wireless sensing system engineering.

The remainder of this paper is organized as follows. Section 2 establishes the sparse mapping, MIMO reception, and equivalent recovery model for sensing short packets. Section 3 presents the optimization objective, construction method, and implementation procedure of LCB-SM. Section 4 introduces the frozen-index set and the proposed FIP-OMP algorithm. Section 5 analyzes the recovery reliability and computational overhead of the proposed methods through simulations and SDR experiments. Finally, Section 6 concludes this paper.

## 2. Design Framework of Low-Complexity Sparse Vector Coding Scheme

**Figure 1:**
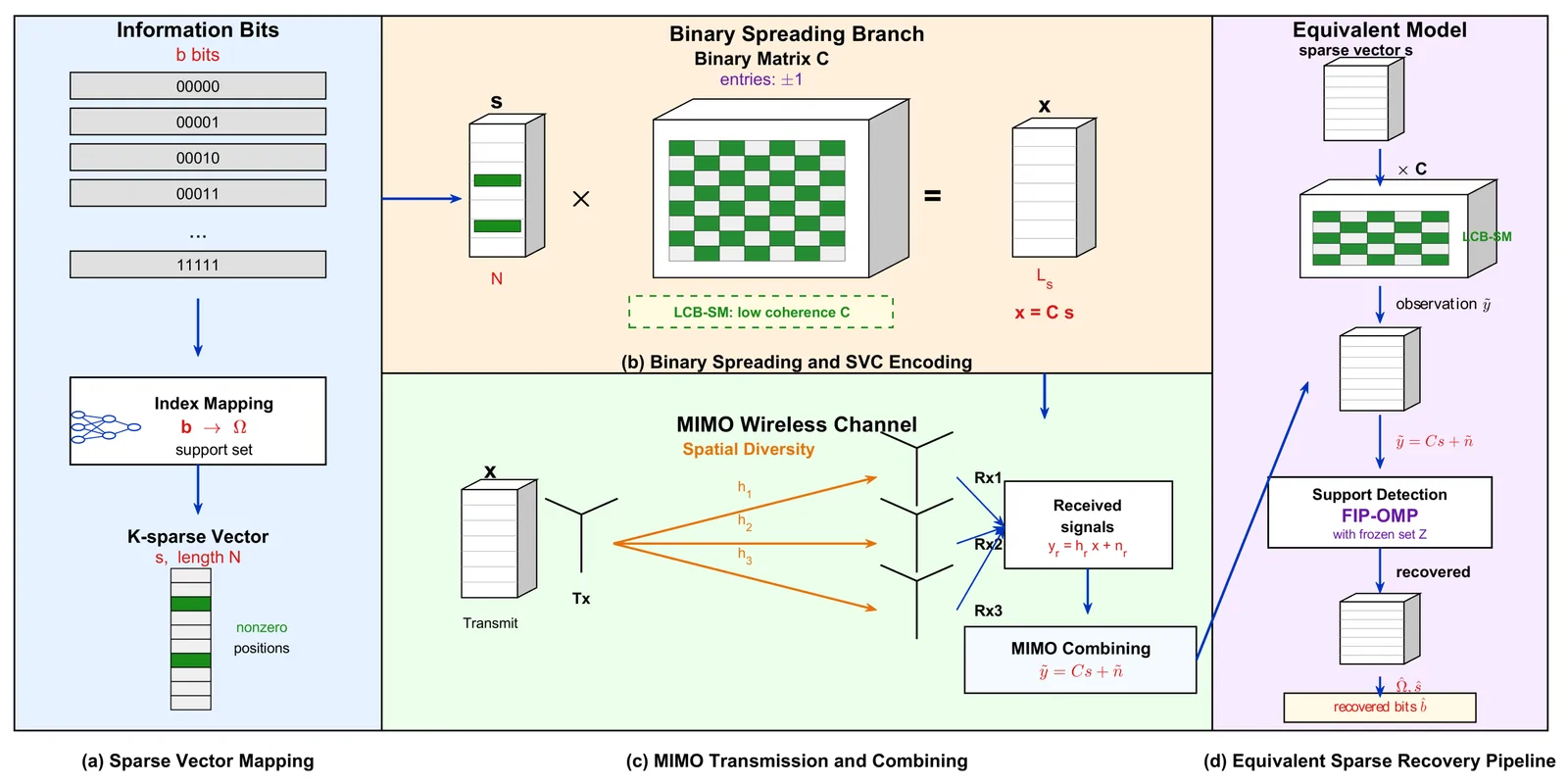
Overall architecture of the proposed MIMO-based sparse vector coding framework for short-packet wireless sensing.

Figure 1 illustrates the LC-SVC for Reliable Short-Packet Transmission in MIMO-based wireless sensor systems. The proposed framework is intended for real-time sensing scenarios such as the tactile internet, robotic teleoperation, and industrial wireless sensing. In these applications, sensory data such as force, tactile information, contact states, or end-effector states are typically generated continuously with short sampling periods and need to be uploaded to an edge controller or a remote processing unit in real time over wireless links. Since such sensory data are directly involved in state estimation, closed-loop feedback, or control decision-making, transmission errors may lead to abrupt feedback variations, incorrect state judgments, or fluctuations in control inputs. Therefore, short-packet links generally need to satisfy stringent requirements for sensing: high reliability, low processing latency, and low terminal complexity.

At the transmitter, the sensor node first acquires continuous physical quantities, such as force, tactile, or contact-state signals. After sampling, quantization, and packetization, each sensing short packet is represented as an information bit sequence of length *b*, denoted by $u\in {\left\{0,1\right\}}^{b}$. Here, *b* denotes the number of effective sensing information bits carried by a single short packet. Unlike conventional direct modulation schemes, LC-SVC first maps the bit sequence to the support set of a sparse vector. Specifically, after index mapping, u is transformed into an *N*-dimensional *K*-sparse vector $s\in R^{N},{∥s∥}_{0}=K,$ where the position set of the nonzero elements, Ω=supp(s), is used to carry the sensing short-packet information.

(1) b≤log2NK.

After sparse mapping, the sensor node further performs binary spreading encoding on the sparse vector. Let the binary spreading matrix be $C\in {\left\{+1,-1\right\}}^{L_{s}\times N},$ where $L_{s}$ denotes the spreading length and usually satisfies $L_{s}<N$. The short-packet transmit signal generated at the transmitter is given by

(2) x=Cs.

Since the entries of C take only +1 or −1, the spreading operation in (2) can be implemented through sign flipping and accumulation, without requiring complex multiplication operations. Therefore, this coding method is well suited for deployment on sensor nodes with limited computational capability and energy resources.

In the LC-SVC framework, the inter-column coherence of the spreading matrix C directly affects the sparse support recovery performance at the receiver. If different columns are highly correlated, noise perturbations may cause the receiver to select incorrect atoms during support detection, thereby leading to short-packet recovery failure. To address this issue, this paper adopts an LCB-SM design at the transmitter, referred to as LCB-SM. As shown in the Binary Spreading Branch in Figure 1, LCB-SM reduces the correlation among the columns of the spreading matrix while preserving the ±1 binary structure, thereby providing a more stable measurement basis for subsequent sparse recovery.

During wireless transmission, the transmit signal x propagates through the MIMO-assisted wireless sensing link and arrives at the multi-antenna receiver. This paper considers a receive diversity structure equipped with $N_{r}$ receive antennas, which can be used to improve the equivalent received signal-to-noise ratio of short-packet transmission. For the *r*-th receive antenna, the received signal can be expressed as

(3) $$y_{r}=h_{r}x+n_{r}=h_{r}Cs+n_{r},\;r=1,2,\ldots ,N_{r},$$

where $h_{r}$ denotes the quasi-static fading coefficient between the sensor node and the *r*-th receive antenna, and $n_{r}$ represents the additive white Gaussian noise on the corresponding receive branch. The noise vectors are assumed to be independent across the receive branches and satisfy $n_{r}∼\mathrm{CN}\left(0,\sigma_{n}^{2}I_{L_{s}}\right)$. Since the duration of a sensing short packet is typically much shorter than the channel coherence time, a quasi-static fading model is adopted in this paper to characterize the wireless sensing link [2,41,42].

At the multi-antenna receiving gateway, the channel coefficients can be estimated using pilot symbols transmitted within each short packet. Since the channel is assumed to remain unchanged over one packet, the same channel estimate can be used for the entire packet. To isolate the performance of the proposed spreading-matrix design and sparse recovery algorithm, this paper assumes perfect receiver-side channel state information (CSI), i.e., ${\hat{h}}_{r}=h_{r}$. Under this assumption, maximum-ratio combining (MRC) is applied to obtain the equivalent observation signal as [43]

(4) $$\tilde{y}=\frac{\sum_{r=1}^{N_{r}}h_{r}^{*}y_{r}}{\sum_{r=1}^{N_{r}}{\left|h_{r}\right|}^{2}}=Cs+\tilde{n},$$

where ${\left(\cdot \right)}^{*}$ denotes complex conjugation and $\tilde{n}$ denotes the equivalent noise after MRC combining. Let $h={\left[h_{1},\ldots ,h_{N_{r}}\right]}^{T}$. The equivalent noise after MRC and its conditional distribution are given by

(5) $$\begin{array}{cc} \tilde{n} & =\frac{\sum_{r=1}^{N_{r}}h_{r}^{*}n_{r}}{\sum_{r=1}^{N_{r}}{\left|h_{r}\right|}^{2}},\\ \tilde{n}|h & ∼\mathrm{CN}\left(0,\frac{\sigma_{n}^{2}}{\sum_{r=1}^{N_{r}}{\left|h_{r}\right|}^{2}}I_{L_{s}}\right). \end{array}$$

Therefore, although the fading coefficients are normalized out of the desired signal term in (4), their effect does not disappear. Instead, the channel gains determine the equivalent noise power and the instantaneous post-combining SNR through $\sum_{r=1}^{N_{r}}{\left|h_{r}\right|}^{2}$. Consequently, (4) is an equivalent fading-dependent observation model rather than a fixed-SNR pure AWGN channel.

It should be noted that (4) represents an ideal-CSI performance model. In practical short-packet transmission, pilot symbols occupy part of the limited packet length, while channel estimation errors may reduce the coherent combining gain of MRC. The pilot overhead and channel estimation error are not included in the present evaluation because this work focuses on the spreading-matrix construction and the subsequent sparse recovery process. Therefore, the reported results characterize the performance of the proposed LC-SVC scheme under ideal receiver-side CSI.

Moreover, the current receive-diversity model does not explicitly account for near-field propagation or spatial non-stationarity in extremely large antenna arrays [44,45]. Practical parameter estimation in large-scale MIMO arrays may also be affected by array calibration errors, such as unknown gain-phase errors, which further complicate CSI acquisition and array-domain signal processing [46]. For such scenarios, hierarchical channel estimation with candidate pre-selection and multi-level dynamic thresholding can be employed to reduce the estimation search space and improve robustness under different SNR conditions [45]. Joint consideration of practical channel estimation and LC-SVC decoding is left for future work. As can be observed from (4), the MIMO-based receiving structure mainly provides spatial diversity gain, while the backend detection problem after combining still retains the standard SVC sparse recovery form. Therefore, the system can introduce multi-antenna receive gain to improve the reliability of sensing short-packet links without changing the sparse coding structure.

The core task of the receiver is to accurately recover the support set of the sparse vector s given the spreading matrix C and the equivalent observation signal $\tilde{y}$. This process can be expressed as

(6) $$\hat{\Omega }=\mathrm{SupportDetection}\left(\tilde{y},C\right),$$

where $\hat{\Omega }$ denotes the estimated support set. Subsequently, the receiver reconstructs the sparse vector $\hat{s}$ according to $\hat{\Omega }$ and recovers the sensing short-packet bits $\hat{u}$ through inverse index mapping. For real-time scenarios such as tactile feedback, force-control interaction, and industrial state monitoring, support detection errors directly lead to sensing data recovery errors. Therefore, the reliability of support recovery is a key aspect in the receiver design of LC-SVC.

To further exploit the redundant support combinations existing in the SVC mapping process, this paper adopts FIP-OMP at the receiver. Specifically, when NK>2b, there exist redundant regions in the sparse support combination space that are not used by valid information bits. FIP-OMP defines the indices corresponding to these invalid mapping regions as the frozen-index set and prunes them during the atom selection stage of OMP, thereby reducing the probability of noise-induced false detection of invalid indices. In this way, LC-SVC provides a low-coherence binary spreading matrix through LCB-SM at the transmitter and introduces mapping-prior constraints through FIP-OMP at the receiver. These two components jointly improve the reliability and real-time recovery performance of short-packet wireless sensing links.

## 3. Low-Coherence Binary Spreading Matrix-Based Encoding Algorithm

In the MIMO-based wireless sensor systems, the original sensing information is first mapped into a sparse vector, which is then compressed and spread through a spreading matrix for transmission. Since the receiver needs to recover the support set of the sparse vector from the received signal, the distinguishability among different columns of the spreading matrix directly affects the sparse recovery performance. If the inter-column correlation of the spreading matrix is high, OMP-type recovery algorithms are more susceptible to noise perturbations during atom matching, which may lead to the selection of incorrect support positions and consequently increase the block error rate. Therefore, designing a spreading matrix with low coherence is critical for improving the reliability of the SVC system.

To reduce inter-column correlation while maintaining low hardware implementation complexity, this paper adopts LCB-SM. The entries of this matrix are constrained to {+1,−1}; therefore, the spreading operation at the transmitter can be implemented through sign flipping and accumulation, without requiring complex multiplication operations. On the other hand, LCB-SM reduces the probability of confusion among different support positions by controlling the maximum correlation between the columns of the spreading matrix, thereby improving the stability of subsequent sparse vector recovery.

**Figure 2:**
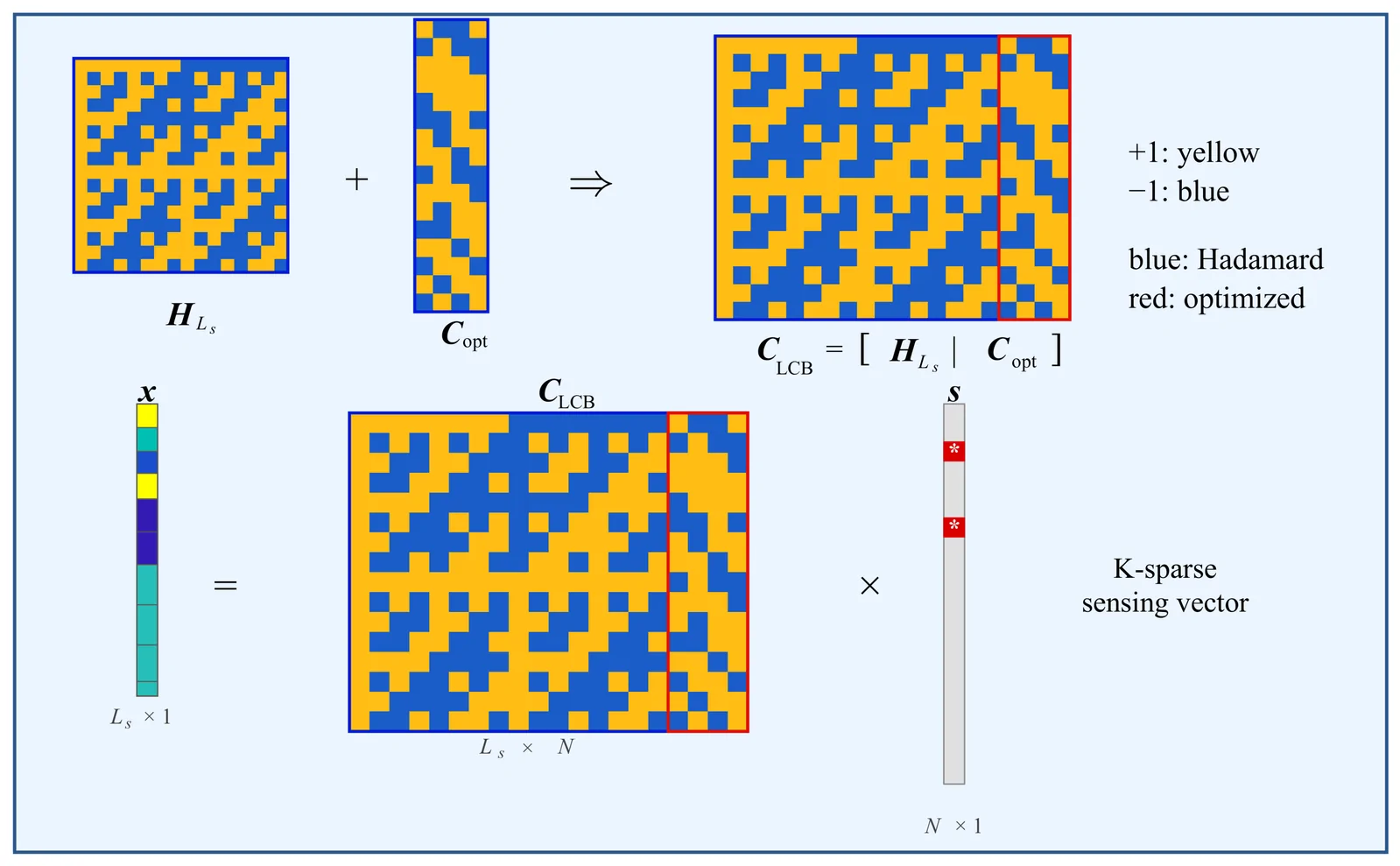
Construction procedure of the proposed LCB-SM. Yellow and blue cells represent +1 and −1 matrix entries, respectively. The blue frame denotes the Hadamard-based portion, whereas the red frame highlights the optimized appended columns. The asterisks mark the positions of the *K* nonzero entries in the *K*-sparse sensing vector s.

Figure 2 illustrates the construction procedure of the proposed LCB-SM. As can be observed, the proposed method first constructs an initial column set using a Hadamard matrix. Since the columns of a Hadamard matrix are pairwise orthogonal, it can provide a zero-coherence initial basis for the spreading matrix. When the target sparse vector dimension *N* is larger than the spreading length $L_{s}$, the Hadamard matrix alone cannot provide a sufficient number of columns. Therefore, the remaining $N-L_{s}$ binary columns need to be further constructed. For each new column to be constructed, LCB-SM calculates its correlation with the currently constructed column set and selects the candidate column that minimizes the maximum correlation. Through this column-by-column extension strategy, the matrix progressively suppresses the overall inter-column coherence while preserving the ±1 binary structure.

Let the binary spreading matrix be given by $C\in {\left\{+1,-1\right\}}^{L_{s}\times N}$. Here, $L_{s}$ denotes the spreading length, and *N* denotes the dimension of the sparse vector. To characterize the similarity among different columns of the spreading matrix, the mutual coherence of matrix C is defined as [31]

(7) $$\mu \left(C\right)=\max_{1\leq i\neq j\leq N}\frac{\left|\langle c_{i},c_{j}\rangle \right|}{{∥c_{i}∥}_{2}{∥c_{j}∥}_{2}}.$$

where $c_{i}$ and $c_{j}$ denote the *i*-th and *j*-th columns of matrix C, respectively, 〈·,·〉 denotes the inner product operation, and ${∥\cdot ∥}_{2}$ denotes the Euclidean norm. A smaller mutual coherence coefficient μ(C) indicates lower similarity among the columns of the matrix, which makes it easier for the receiver to distinguish different sparse positions during support set detection.

The construction of LCB-SM starts from a Hadamard matrix. Let

(8) $$H_{L_{s}}\in {\left\{+1,-1\right\}}^{L_{s}\times L_{s}}$$

denote a Hadamard matrix of order $L_{s}$. Then, the first $L_{s}$ columns of the spreading matrix C are initialized as

(9) $$C_{1:L_{s}}=H_{L_{s}}.$$

Since the columns of the Hadamard matrix are mutually orthogonal, this initialization provides a low-coherence initial column set for subsequent matrix extension. However, when $N>L_{s}$, the number of columns provided by the Hadamard matrix is insufficient to meet the requirements of sparse vector coding. Therefore, the remaining columns need to be further constructed under the binary constraint.

For the *k*-th column to be constructed, where $k>L_{s}$, assume that the currently constructed column set is given by $\left\{c_{1},c_{2},\ldots ,c_{k-1}\right\}$. The objective of LCB-SM is to select, from all possible binary candidate columns, a column that is least similar to the existing column set. Specifically, the selected column should minimize the maximum absolute correlation with the existing columns. Therefore, the construction problem of the *k*-th column can be formulated as

(10) $$c_{k}^{⋆}=\arg\min_{c\in {\left\{+1,-1\right\}}^{L_{s}}}\left(\max_{1\leq j<k}\left|\langle c,c_{j}\rangle \right|\right).$$

Equation (10) shows that LCB-SM is a column-wise implementation of the original min–max design objective. Instead of solving a global optimization over the entire matrix, the construction is carried out sequentially column by column, so that each newly added column minimizes the worst-case correlation with the previously constructed set.

To facilitate the solution of (10), an auxiliary variable $T^{\left(k\right)}$ is introduced to denote the maximum correlation threshold allowed during the construction of the *k*-th column. Then, the construction problem of the *k*-th column can be transformed into the following binary integer optimization problem:

(11) $$\begin{array}{cc} \min_{c,T^{\left(k\right)}}\;\; & T^{\left(k\right)}\\ s.t.\;\; & -T^{\left(k\right)}\leq \langle c,c_{j}\rangle \leq T^{\left(k\right)},\;j=1,2,\ldots ,k-1,\\ \; & c\in {\left\{-1,+1\right\}}^{L_{s}}. \end{array}$$

To convert the bipolar binary variables into standard 0–1 integer variables, the following linear transformation is introduced:

(12) $$c=2m-1,\;m\in {\left\{0,1\right\}}^{L_{s}},$$

where 1 denotes the all-one vector. By substituting (12) into (11), the correlation constraints can be written as

(13) $$\begin{array}{cc} \; & \langle 2m-1,c_{j}\rangle -T^{\left(k\right)}\leq 0,\\ \; & -\langle 2m-1,c_{j}\rangle -T^{\left(k\right)}\leq 0,\;j=1,2,\ldots ,k-1. \end{array}$$

Following the integer-programming formulation, the inequality constraints are incorporated into the objective function through the indicator function I(x), where I(x)=0 for x≤0 and I(x)=+∞ otherwise. Since I(x) is nondifferentiable, it is approximated by the logarithmic barrier function

(14) $$I_{t}\left(x\right)=\left\{\begin{array}{cc} -\frac{1}{t}\log\left(-x\right), & x<0,\\ 0, & x=0,\\ +\infty , & x>0, \end{array}\right.$$

where *t* is the barrier parameter. Following the implementation adopted in the matrix construction, *t* is fixed at 100.

For a fixed correlation threshold $T^{\left(k\right)}$, the objective function for constructing the *k*-th column is

(15) $$\begin{array}{cc} f^{\left(k\right)}\left(m;T^{\left(k\right)}\right)= & \sum_{j=1}^{k-1}I_{t}\;\left(\langle 2m-1,c_{j}\rangle -T^{\left(k\right)}\right)\\ \; & +\sum_{j=1}^{k-1}I_{t}\;\left(-\langle 2m-1,c_{j}\rangle -T^{\left(k\right)}\right). \end{array}$$

For candidates strictly inside the feasible region, the finite part of (15) can equivalently be expressed as

(16) $$\begin{array}{cc} f^{\left(k\right)}\left(m;T^{\left(k\right)}\right)= & -\frac{1}{t}\sum_{j=1}^{k-1}\log\;\left(T^{\left(k\right)}-\langle 2m-1,c_{j}\rangle \right)\\ \; & -\frac{1}{t}\sum_{j=1}^{k-1}\log\;\left(T^{\left(k\right)}+\langle 2m-1,c_{j}\rangle \right). \end{array}$$

The binary vector used to construct the new column is obtained by

(17) $$m^{⋆}=\arg\min_{m\in {\left\{0,1\right\}}^{L_{s}}}f^{\left(k\right)}\left(m;T^{\left(k\right)}\right).$$

At a fixed $T^{\left(k\right)}$, an infeasible candidate produces an infinite objective value and is discarded during implicit enumeration, whereas the candidate minimizing the finite barrier objective is retained. If all candidate vectors produce infinite objective values, no feasible column exists under the current threshold, and $T^{\left(k\right)}$ is increased by one.

Once a finite minimizer is obtained, the new column is constructed as $c_{k}=2m^{⋆}-1$. The threshold found for the current column is inherited by the next construction step, since the minimum achievable maximum correlation is nondecreasing as the number of constructed columns increases. This relationship between the barrier objective and the threshold update is explicitly implemented in Algorithm 1.

The threshold search in Algorithm 1 terminates in a finite number of steps for the finite-dimensional binary design space considered here. For a bipolar column of length $L_{s}$, the absolute inner product between two candidate columns is upper bounded by $L_{s}$. Therefore, once $T^{\left(k\right)}$ increases from zero to $L_{s}$, all candidate columns satisfy the threshold constraint. Since the candidate set contains at most $2^{L_{s}}$ binary vectors, the implicit enumeration at each threshold is also finite.

The barrier-based threshold search and column construction are performed offline only once. At each threshold, implicit enumeration examines $2^{L_{s}}$ binary candidates at most. Evaluating one candidate requires correlations with the k−1 previously constructed columns, giving a worst-case complexity of $O(2^{L_{s}}\left(k-1\right)L_{s})$ for constructing the *k*-th column at a fixed threshold. If $q_{k}$ threshold values are examined, the total worst-case offline complexity is $O\;\left(2^{L_{s}}L_{s}\sum_{k=L_{s}+1}^{N}q_{k}\left(k-1\right)\right)$, while the storage complexity of the resulting matrix is $O(L_{s}N)$.

Although the offline construction cost increases exponentially with $L_{s}$, the matrix is generated only once and stored at both the transmitter and receiver. Therefore, it does not affect the online transmission latency. Since s contains only *K* nonzero entries and the entries of C are restricted to ±1, online spreading requires approximately $O(KL_{s})$ additions and subtractions without complex multiplications. For much larger spreading dimensions, more scalable search strategies may be incorporated into the same correlation-constrained design framework.

After all columns have been constructed, the spreading matrix is obtained as

(18) $$C=[c_{1},c_{2},\ldots ,c_{N}].$$

As described in (2), the constructed LCB-SM is used to spread the sparse vector and generate the SVC codeword. At the multi-antenna gateway of the considered MIMO-based wireless sensor system, signal combining is performed to obtain the equivalent observation model (4). The optimization target of LCB-SM is still the spreading matrix ***C*** involved in the sparse recovery process. Therefore, the proposed matrix design method is independent of the specific antenna configuration and can be directly applied to single-antenna links, receive-diversity links, and MIMO-based wireless sensor systems.
**Algorithm 1** LCB-SM Binary Spreading Matrix Construction  1:**Input:** Spreading length $L_{s}$, sparse-vector dimension *N*, and Hadamard matrix $H_{L_{s}}$  2:**Output:** Binary spreading matrix $C\in {\left\{+1,-1\right\}}^{L_{s}\times N}$  3:Initialize $C_{1:L_{s}}=H_{L_{s}}$  4:Set t=100 and $T^{\left(L_{s}\right)}=0$  5:**for** $k=L_{s}+1$ to *N* **do**  6:    Set $T^{\left(k\right)}=T^{\left(k-1\right)}$   7:   **repeat**  8:       Compute$$f_{\min}^{\left(k\right)}=\min_{m\in {\left\{0,1\right\}}^{L_{s}}}f^{\left(k\right)}\left(m;T^{\left(k\right)}\right)$$
       by implicit enumeration  9:       **if** $f_{\min}^{\left(k\right)}<+\infty$ **then**10:          Let $m^{⋆}$ be a minimizer of $f^{\left(k\right)}\left(m;T^{\left(k\right)}\right)$11:          Set $c_{k}=2m^{⋆}-1$12:          **break**13:       **else**14:          Update $T^{\left(k\right)}\leftarrow T^{\left(k\right)}+1$15:       **end if**16:    **until** $f_{\min}^{\left(k\right)}<+\infty$17:**end for**18:**return** $C=[c_{1},c_{2},\ldots ,c_{N}]$

## 4. Frozen-Index-Pruned OMP-Based Decoding Algorithm

The LCB-SM designed in the previous section reduces the inter-column coherence of the spreading matrix, thereby improving the stability of sparse recovery. However, conventional OMP still performs greedy atom selection over the complete index space and does not exploit the structured redundancy of SVC mapping. To address this limitation, this paper proposes a frozen-index-pruned orthogonal matching pursuit algorithm, termed FIP-OMP, which prevents indices that cannot appear in valid transmitted sparse vectors from participating in atom selection.

For an *N*-dimensional *K*-sparse vector, the number of all possible support combinations is

(19) M=NK.

A *b*-bit information sequence contains $2^{b}$ possible information states. Assuming $2^{b}\leq M$, the number of unused support combinations is

(20) Ggap=NK−2b.

It should be emphasized that an unused support is a complete *K*-element index combination, whereas a frozen index is a single coordinate that does not occur in any valid support.

To establish a deterministic relationship between information sequences and support combinations, the smallest active index range is defined as

(21) P⋆=minp∈{K,…,N}:pK≥2b.

Let U(P⋆,K)={U0,…,UP⋆K−1} denote all *K*-element subsets of $\left\{1,\ldots ,P^{⋆}\right\}$, arranged in lexicographic order. The valid support codebook is defined as

$$V=\{U_{0},\ldots ,U_{2^{b}-1}\}.$$

For an information sequence $u=(u_{b-1},\ldots ,u_{0})$, its decimal value is $q=\sum_{\ell =0}^{b-1}u_{\ell }2^{\ell }\in \left\{0,\ldots ,2^{b}-1\right\}$, and the corresponding support is determined by $\phi \left(u\right)=U_{q}$. This deterministic mapping rule is fixed and shared by the transmitter and receiver.

Accordingly, the complete, active, and frozen-index sets are defined as

$$I=\left\{1,\ldots ,N\right\},\;A=\left\{1,\ldots ,P^{⋆}\right\},\;Z=\left\{P^{⋆}+1,\ldots ,N\right\},$$

respectively. Every valid support satisfies ϕ(u)⊆A, and hence $s_{j}=0$ for all j∈Z. The total mapping redundancy can be decomposed as

(22) NK−2b︸Ggap=NK−P⋆K︸GZ+P⋆K−2b︸GA,

where $G_{Z}$ denotes the unused combinations containing at least one frozen index, while $G_{A}$ denotes the remaining unused combinations located entirely within the active set.

For the configuration N=16, K=2, and b=6, we have 162=120 and $2^{6}=64$, resulting in $G_{\mathrm{gap}}=56$. Since 112=55<64 and 122=66≥64, we obtain $P^{⋆}=12$, A={1,…,12}, and Z={13,…,16}. Among the 56 unused combinations,

GZ=162−122=54

contain at least one frozen index, giving a frozen-index coverage ratio of $G_{Z}/G_{\mathrm{gap}}=96.43\%$. The remaining two unused supports, {10,12} and {11,12}, are located entirely within A and cannot be removed by freezing individual indices.

When $P^{⋆}=N$, the frozen set is empty and FIP-OMP reduces to conventional OMP. In particular, if NK=2b, no mapping redundancy exists. If NK>2b but $P^{⋆}=N$, unused support combinations still exist, but no individual coordinate can be globally frozen without removing a valid information state. In this case, no frozen-index pruning gain is claimed.

**Figure 3:**
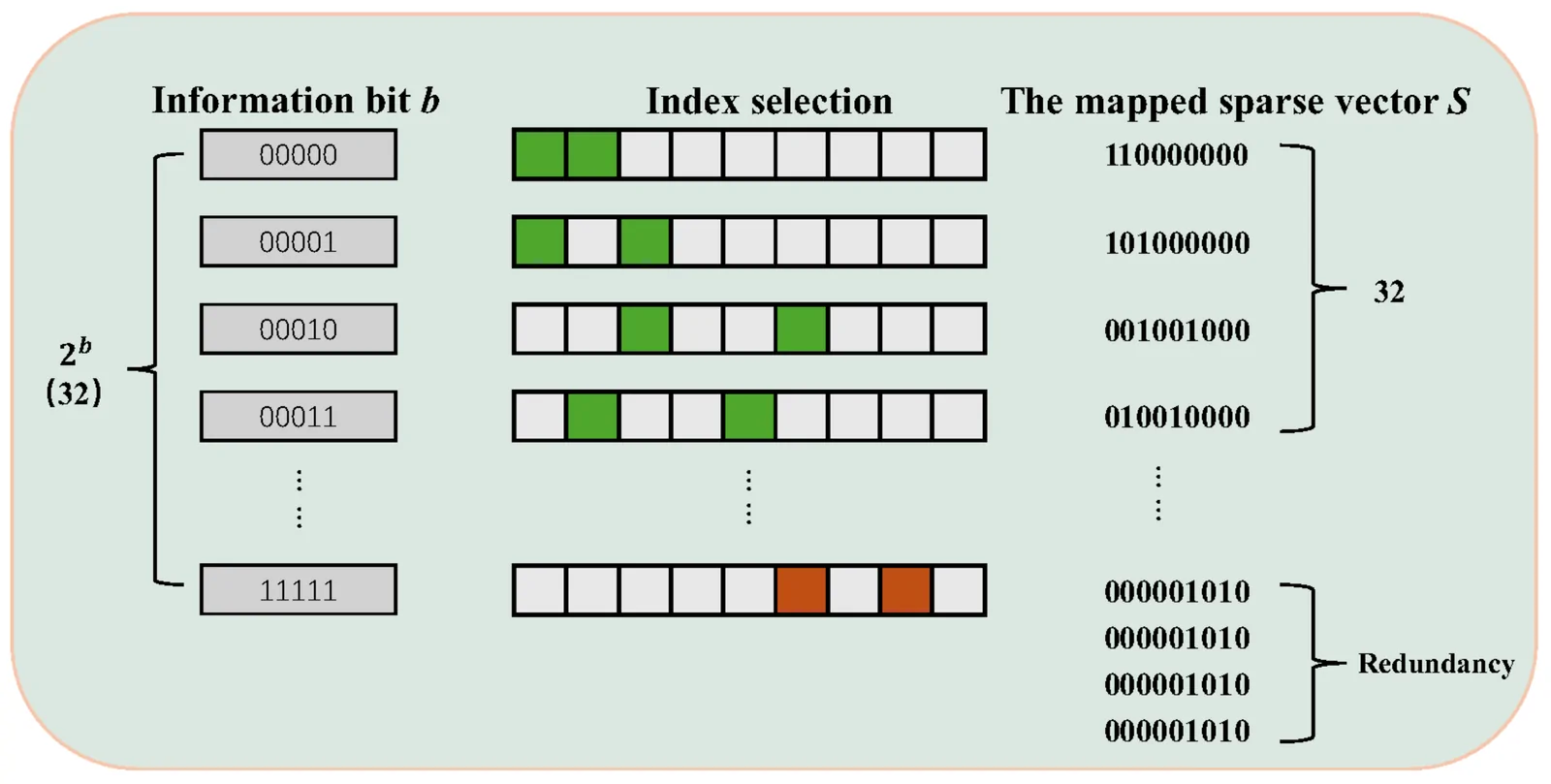
Illustration of SVC mapping redundancy and the frozen-index pruning mechanism in FIP-OMP. The ellipses indicate omitted intermediate mapping cases. Green blocks represent the selected nonzero indices in valid mappings, whereas orange blocks highlight the indices associated with the boundary mapping used to identify the subsequent redundant combinations and construct the frozen-index set.

Figure 3 illustrates the deterministic support mapping, the decomposition of the unused combinations, and the frozen-index pruning mechanism of FIP-OMP. The left part represents the input information sequences, the middle part shows the valid support mapping, and the right part presents the corresponding sparse vectors and restricted atom selection region.

FIP-OMP introduces a frozen-index masking operation into the conventional OMP iteration. Let $r_{t-1}$ denote the residual at the beginning of the *t*-th iteration. The correlation vector is first calculated as

(23) $$g^{\left(t\right)}=C^{H}r_{t-1}.$$

The correlation values corresponding to frozen indices are then masked as

(24) $${\tilde{g}}_{j}^{\left(t\right)}=\left\{\begin{array}{cc} 0,\; & j\in Z,\\ g_{j}^{\left(t\right)},\; & j\in A. \end{array}\right.$$

Therefore, even if noise produces a large correlation peak at a frozen index, that index cannot participate in the subsequent atom selection step.

Based on the masked correlation vector, the atom selected at the *t*-th iteration is

(25) $$\lambda_{t}=\arg\max_{j\in A\setminus \Lambda_{t-1}}\left|{\tilde{g}}_{j}^{\left(t\right)}\right|,$$

where $\Lambda_{t-1}$ denotes the support estimated during the previous iterations. The support is updated as

(26) $$\Lambda_{t}=\Lambda_{t-1}\cup \left\{\lambda_{t}\right\}.$$

After obtaining $\Lambda_{t}$, the nonzero coefficients are estimated by

(27) $${\hat{\theta }}_{t}=\arg\min_{\theta }{∥\tilde{y}-C_{\Lambda_{t}}\theta ∥}_{2}^{2},$$

where $C_{\Lambda_{t}}$ denotes the submatrix composed of the columns whose indices belong to $\Lambda_{t}$. The residual is then updated as

(28) $$r_{t}=\tilde{y}-C_{\Lambda_{t}}{\hat{\theta }}_{t}.$$

The above procedure is repeated for *K* iterations to obtain the recovered support $\Lambda_{K}$ and the reconstructed sparse vector $\hat{s}$. The complete decoding procedure is summarized in Algorithm 2.

If $\Lambda_{K}\in V$, the corresponding information sequence is obtained through the inverse mapping $\phi^{-1}\left(\Lambda_{K}\right)$. If $\Lambda_{K}\notin V$, the recovered support corresponds to one of the $G_{A}$ unused combinations inside the active set, and the packet is declared incorrectly decoded.

**Algorithm 2** Proposed FIP-OMP Decoding Algorithm
  1:**Input:** Observation y, matrix C, sparsity *K*, frozen set Z.  2:**Initialize:** Residual $r_{0}=y$, support set $\Lambda_{0}=Ø$, t=1.  3:**while** t≤K **do**  4:   Compute correlation: $g^{\left(t\right)}=C^{T}r_{t-1}$;  5:   **Masking:** ${\tilde{g}}_{j}^{\left(t\right)}=g_{j}^{\left(t\right)}\cdot I\left\{j\notin Z\right\}$ for all j∈{1,…,N};  6:   **Atom Selection:** $\lambda_{t}=\arg\max_{j\notin \Lambda_{t-1}}\left|{\tilde{g}}_{j}^{\left(t\right)}\right|$;  7:   **Update Support:** $\Lambda_{t}=\Lambda_{t-1}\cup \left\{\lambda_{t}\right\}$;  8:   **LS Estimation:** $\theta_{t}=\arg\min_{x}{∥y-C_{\Lambda_{t}}x∥}_{2}$;  9:   **Update Residual:** $r_{t}=y-C_{\Lambda_{t}}\theta_{t}$;10:   t=t+1;11:
**end while**
12:**Output:** Reconstructed support $\Lambda_{K}$ and signal $\hat{s}$.


Having described the iterative recovery procedure, we next establish a sufficient condition under which FIP-OMP can exactly recover the true support set.

### Recovery Condition of FIP-OMP

Let $C_{A}$ denote the submatrix formed by the columns of C whose indices belong to A. Its effective mutual coherence is defined as

(29) $$\mu_{A}=\max_{\begin{array}{c} i,j\in A\\ i\neq j \end{array}}\frac{\left|\langle c_{i},c_{j}\rangle \right|}{{∥c_{i}∥}_{2}{∥c_{j}∥}_{2}}.$$

Since every column of the binary spreading matrix contains $L_{s}$ entries from {+1,−1}, all columns have the same Euclidean norm $\sqrt{L_{s}}$.

**Proposition 1.** 
*Consider the noiseless observation $\tilde{y}=Cs$, where the true transmitted support satisfies Ω∈V, and hence Ω⊆A and |Ω|=K. If*

(30) $$\mu_{A}<\frac{1}{2K-1},$$

*then FIP-OMP exactly recoversΩ within K iterations.*

**Proof.**  The frozen-index masking operation prevents every index in Z from participating in atom selection. Therefore, FIP-OMP is equivalent to applying conventional OMP to the reduced matrix $C_{A}$. According to the standard mutual-coherence-based recovery condition of OMP [47], a *K*-sparse signal can be exactly recovered when $\mu_{A}<1/\left(2K-1\right)$. Hence, the stated result follows. □

Because A⊆I, the maximization used to calculate $\mu_{A}$ is performed over a subset of the column pairs used to calculate μ(C). Consequently,

(31) $$\mu_{A}\leq \mu \left(C\right).$$

Conventional OMP requires the coherence condition to hold over all columns of C, whereas FIP-OMP only requires it to hold over the active columns in $C_{A}$. If every column pair attaining μ(C) contains at least one frozen index, then $\mu_{A}<\mu \left(C\right)$, and frozen-index pruning strictly relaxes the sufficient recovery condition. Otherwise, $\mu_{A}$ may equal μ(C), and no strict theoretical improvement is claimed. For the main FIP-OMP BLER evaluation with K=2, the sufficient recovery condition becomes $\mu_{A}<1/3$.

Under noisy conditions, the mutual-coherence condition alone does not guarantee exact recovery because the result also depends on the noise level and the minimum nonzero coefficient magnitude. Nevertheless, frozen indices cannot be selected by FIP-OMP, thereby eliminating one source of noise-induced support errors. The $G_{A}$ unused supports located entirely within the active set, however, remain possible recovery outputs.

Having established the sufficient recovery condition, we next examine the computational complexity of FIP-OMP.

The full correlation calculation in (23) requires $O(L_{s}N)$ operations per iteration, while frozen-index masking requires an additional O(N) operations. Over *K* iterations, the correlation and masking costs are $O(KL_{s}N)$ and O(KN), respectively. The least-squares update is identical to that used in conventional OMP. Therefore, FIP-OMP has an overall dominant complexity of

$$O(KL_{s}N+KN),$$

which remains in the same asymptotic order as conventional OMP and does not require the multiple candidate paths used by MMP.

In summary, the deterministic support mapping converts part of the unused support space into an explicit frozen-index prior. FIP-OMP then prevents frozen indices from participating in atom selection while retaining every valid transmitted support. LCB-SM reduces the coherence of the complete spreading matrix, whereas FIP-OMP restricts recovery to the valid index region. These two mechanisms jointly improve the conditions for reliable sparse support recovery while maintaining low decoding complexity.

## 5. Performance Evaluation and Experimental Validation

To verify the effectiveness of the proposed LC-SVC architecture for wireless sensing short-packet transmission, this section evaluates its performance from both simulation and experimental perspectives. First, under quasi-static fading channels, the inter-correlation suppression capability of the LCB-SM at the transmitter and the recovery reliability of the FIP-OMP decoder at the receiver are evaluated, so as to characterize the block recovery performance of sensing short packets over wireless links. Subsequently, the average running time of different decoding algorithms is measured to verify their applicability to real-time sensing systems. Finally, a force-feedback sensing data transmission experiment is established based on a SDR platform. Through waveform reconstruction of real tactile sensor data and root mean square error (RMSE) evaluation, the engineering feasibility of the proposed LC-SVC architecture in practical wireless sensing scenarios is validated.

### 5.1. Simulation Setup

This section summarizes the common simulation settings used to evaluate the proposed LC-SVC architecture from three aspects: matrix-level reliability, decoder-level reliability–complexity tradeoff, and joint system-level performance.

The simulations are conducted over quasi-static Rayleigh fading channels with additive white Gaussian noise at each receive branch. According to the MIMO-based receiving model in Section 2, all results are obtained after multi-antenna combining. The receiver is equipped with $N_{r}=4$ antennas and employs perfect CSI and normalized MRC. The uncertainty of the Monte Carlo BLER estimates is quantified using two-sided 95% Wilson confidence intervals. The horizontal axis is the average pre-MRC SNR per receive branch.

For the matrix-level comparison, the proposed LCB-SM matrix is compared with Bernoulli, Bi-TpCM, and optimized partial hadamard matrix (OPHM) under the same conventional OMP decoder. For the decoder-level comparison, the proposed LCB-SM matrix is fixed and FIP-OMP is compared with OMP, MMP, and CoSaMP. The joint system-level evaluation combines the proposed matrix and decoder. The main simulation parameters are summarized in Table 1.

For each Monte Carlo block, the sparse support is drawn uniformly, the nonzero coefficients follow the unit-energy QPSK alphabet, and the BLER statistics are accumulated over sufficient blocks. For matrix-level comparison, the Bernoulli and OPHM results are averaged over independently generated matrix realizations, and the uncertainty is quantified by two-sided 95% Wilson confidence intervals.

For the decoder-level comparison, the transmitter uses the same LCB-SM matrix, and FIP-OMP is evaluated against OMP, MMP, and CoSaMP under the configurations $\left(N,L_{s},K,b\right)=\left(16,12,2,6\right)$, (24,16,2,7), and (24,16,3,10). Only FIP-OMP uses the active/frozen-index prior, whereas all decoders process the same MRC output.

### 5.2. Effect of Transmitter-Side LCB-SM on the Recovery Reliability of Sensing Short Packets

**Figure 4:**
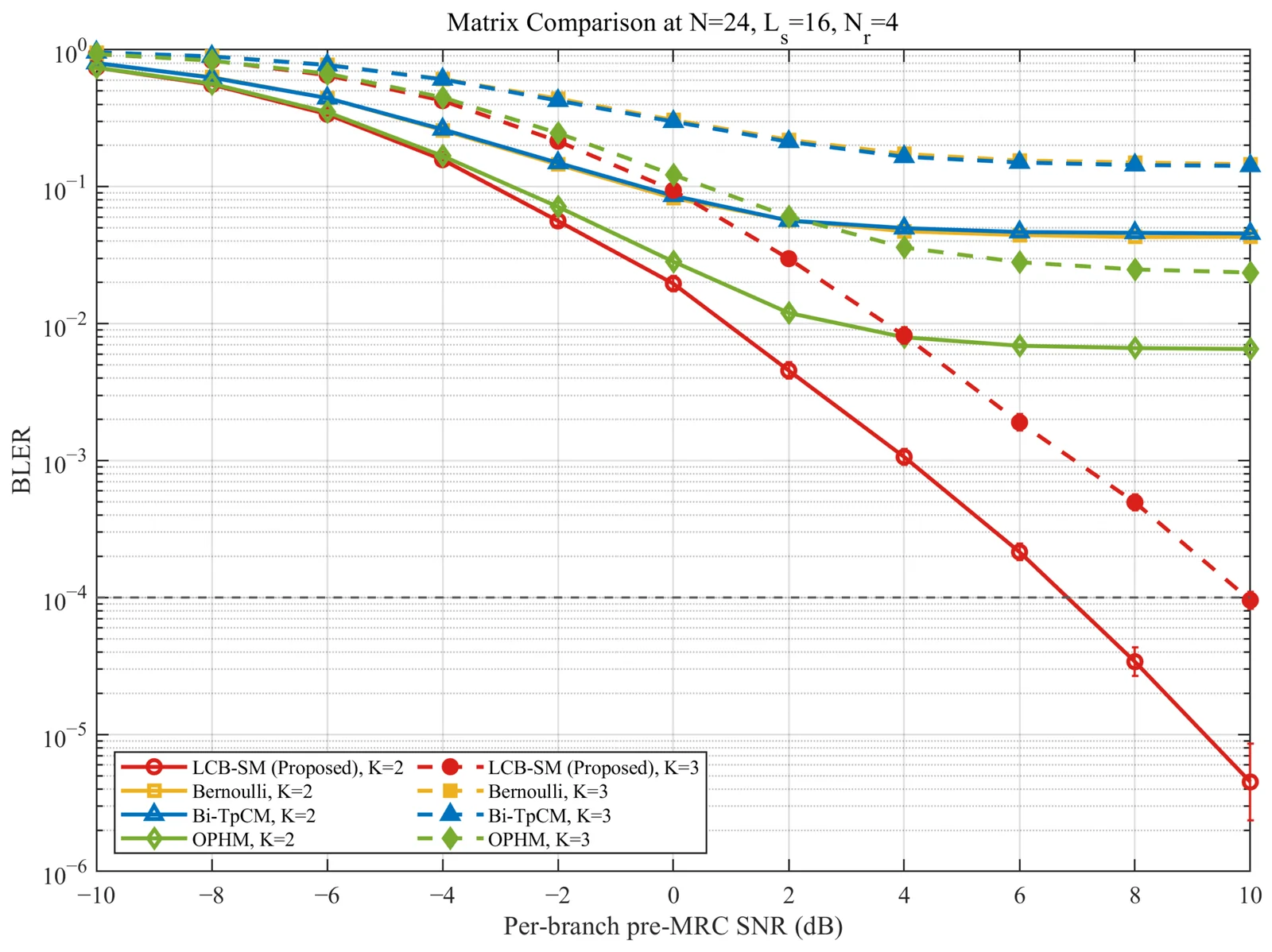
BLER performance comparison of different binary spreading matrices at N=24, $L_{s}=16$, and $N_{r}=4$. Solid curves correspond to K=2, and dashed curves correspond to K=3.

Figure 4 shows the BLER performance comparison of sensing short packets versus the signal-to-noise ratio under different binary spreading matrix schemes. According to the MIMO-based wireless sensing link model established in Section 2, after combining at the multi-antenna receiver, the sparse support set still needs to be recovered within the equivalent SVC detection model. Therefore, the inter-column distinguishability of the spreading matrix directly affects whether the sensing data block can be correctly recovered.

To isolate the effect of the transmitter-side matrix construction, all matrix schemes in this subsection are decoded by the same conventional OMP algorithm. No active-index or frozen-index restriction is imposed. For each Monte Carlo block, the *K*-element support is selected uniformly from {1,…,N}, and the nonzero sparse coefficients are drawn from the unit-energy QPSK alphabet. All binary spreading matrices are normalized by $1/\sqrt{K}$. The receiver employs perfect CSI and normalized MRC over $N_{r}=4$ independent quasi-static Rayleigh fading branches, and the horizontal axis denotes the average pre-MRC SNR per receive branch.

The compared spreading matrices include Bernoulli, Bi-TpCM, OPHM, and the proposed LCB-SM. Bernoulli represents the random SVC baseline, while Bi-TpCM and OPHM represent structured binary matrix constructions. In the revised simulation, OPHM is also included as an additional baseline. The Bernoulli and OPHM results are averaged over independently generated matrix realizations, and the uncertainty is quantified by two-sided 95% Wilson confidence intervals.

As shown in Figure 4, the proposed LCB-SM matrix achieves a lower BLER across different signal-to-noise ratio regions, indicating that it can effectively improve the recovery reliability of short-packet data in MIMO-based sensing links.

For the configuration N=24, $L_{s}=16$, and K=2, LCB-SM exhibits the steepest BLER decrease among the compared schemes. Around the target BLER of $10^{-2}$, LCB-SM requires approximately 3.5 dB pre-MRC SNR, whereas OPHM requires approximately 4.7 dB. This corresponds to an SNR gain of about 1.2 dB over OPHM. In contrast, the Bernoulli and Bi-TpCM matrices remain at BLER levels much higher than $10^{-2}$ in the high-SNR region and therefore do not reach the $10^{-2}$ target within the simulated SNR range. This result indicates that the proposed LCB-SM matrix can achieve the same sensing short-packet recovery reliability with a lower receive-branch SNR.

The high-SNR behavior further demonstrates the benefit of reducing inter-column correlation. When the pre-MRC SNR is 10 dB and K=2, LCB-SM reaches a BLER on the order of $10^{-5}$, whereas OPHM remains around $10^{-2}$ and the Bernoulli and Bi-TpCM matrices exhibit evident error floors at the level of several $10^{-2}$ to $10^{-1}$. Therefore, the gain of LCB-SM is not only reflected by a horizontal SNR shift at a given BLER, but also by its ability to avoid the high-SNR error floor caused by ambiguous spreading columns.

To further examine the sparsity sensitivity, Figure 4 also includes the K=3 case for N=24 and $L_{s}=16$. Compared with the K=2 case, increasing *K* enlarges the number of possible support combinations and makes sparse support recovery more difficult. As a result, the BLERs of all schemes increase. Nevertheless, LCB-SM still maintains a clear reliability advantage. At 10 dB, LCB-SM reaches approximately $10^{-4}$, while OPHM remains around $2.5\times 10^{-2}$ and the Bernoulli and Bi-TpCM matrices remain around $1.5\times 10^{-1}$. Thus, under the tested K=3 configuration, LCB-SM reduces the BLER by more than two orders of magnitude compared with OPHM and by about three orders of magnitude compared with the Bernoulli and Bi-TpCM baselines. This confirms that the proposed matrix construction is not limited to the two-sparse case, but remains effective when more active sensing components are transmitted in one short packet.

**Figure 5:**
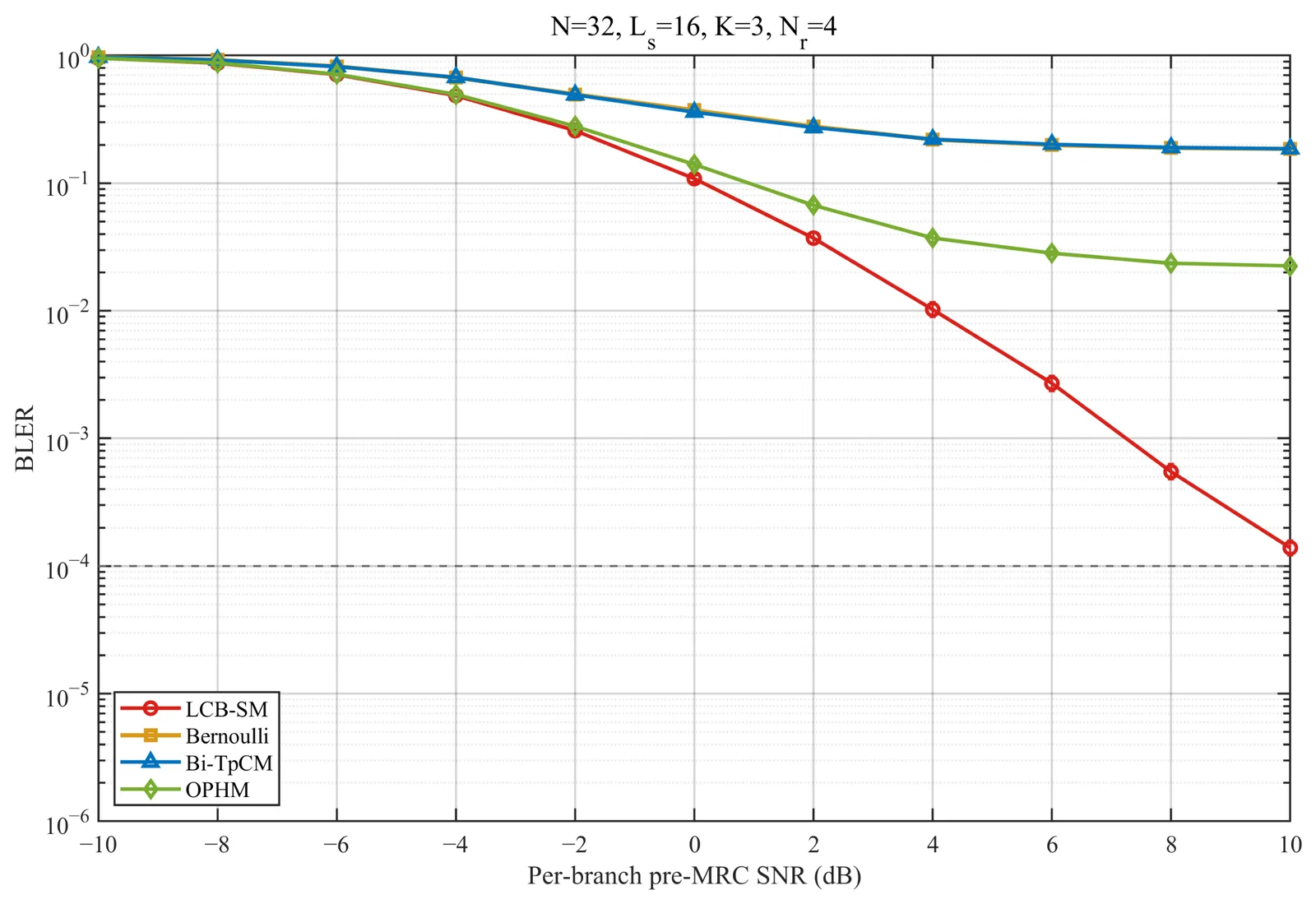
BLER performance comparison of different binary spreading matrices under the larger configuration $\left(N,L_{s},K,b\right)=\left(32,16,3,10\right)$ with $N_{r}=4$, demonstrating the scalability of the proposed LCB-SM matrix.

To further verify the scalability of the proposed matrix construction, we also evaluate the larger configuration $\left(N,L_{s},K,b\right)=\left(32,16,3,10\right)$ under the same receiver setting. Compared with the N=24 cases, this setting enlarges both the candidate-index space and the number of possible support combinations, making sparse recovery more challenging. As shown in Figure 5, LCB-SM still exhibits the steepest BLER decay among the compared matrices. At 10 dB, LCB-SM reaches a BLER of approximately $10^{-4}$, whereas OPHM remains around $2\times 10^{-2}$ and the Bernoulli and Bi-TpCM matrices stay at much higher BLER levels. This result confirms that the proposed low-coherence binary matrix is effective not only in the moderate-size configurations, but also in a larger sparse recovery setting with more active components.

The above performance improvement mainly originates from the improved inter-column correlation characteristics of the LCB-SM matrix. For SVC mapping, each column of the spreading matrix corresponds to a candidate sparse index atom, which can also be interpreted as the physical-layer projection feature of different quantized sensing-state combinations. When two columns have a high normalized correlation, their matched-filter outputs become difficult to distinguish after fading, MRC combining, and noise perturbation. This may lead OMP to select an incorrect atom, and the resulting support error causes a block error.

**Figure 6:**
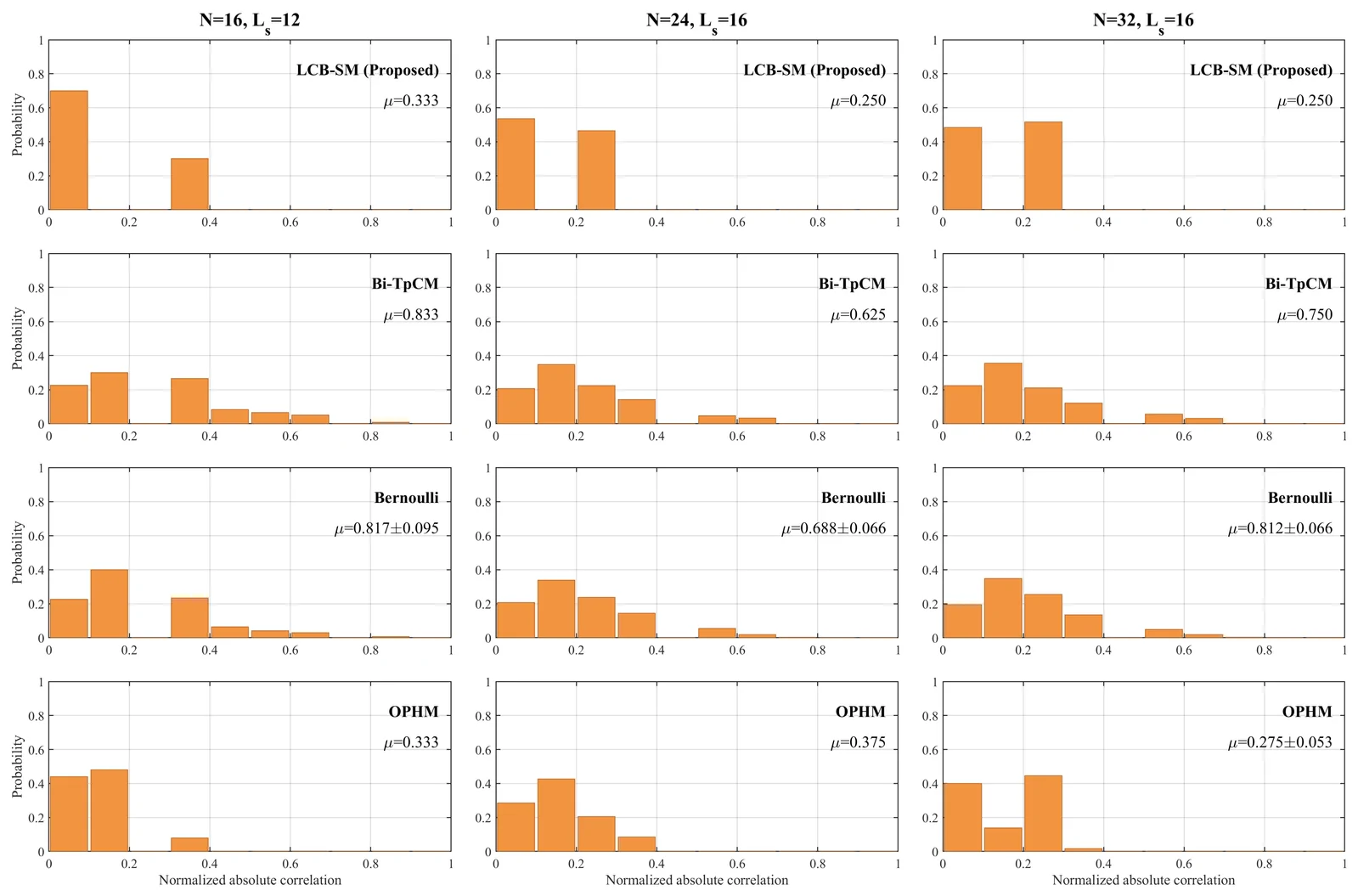
Normalized absolute inter-column correlation distributions of different binary spreading matrices under representative matrix dimensions. The reported μ denotes the maximum normalized absolute inter-column correlation.

To explain the BLER behavior from the matrix-structure perspective, Figure 6 reports the normalized absolute inter-column correlation distributions of different binary spreading matrices. For N=16 and $L_{s}=12$, the proposed LCB-SM has a maximum correlation of μ=0.333, whereas Bi-TpCM and Bernoulli reach μ=0.833 and μ=0.817±0.095, respectively. Although OPHM also obtains μ=0.333, its correlation distribution is less favorable than that of LCB-SM. This explains the better OMP support recovery observed in the BLER comparison.

For N=24 and $L_{s}=16$, LCB-SM further reduces the maximum correlation to μ=0.250, compared with μ=0.625 for Bi-TpCM, μ=0.688±0.066 for Bernoulli, and μ=0.375 for OPHM. For N=32 and $L_{s}=16$, LCB-SM still maintains μ=0.250, while Bi-TpCM and Bernoulli increase to μ=0.750 and μ=0.812±0.066, respectively. These results confirm that LCB-SM remains effective as the candidate-index space grows.

The histograms in Figure 6 also show that Bernoulli and Bi-TpCM contain a visible proportion of high-correlation column pairs, especially above 0.5, whereas LCB-SM concentrates more column pairs in the low-correlation region. This structural difference makes the candidate support indices easier to distinguish and reduces incorrect atom selection during OMP matching.

Overall, the matrix-level results show that the proposed LCB-SM improves sensing short-packet recovery by suppressing inter-column correlation. Under the common OMP decoder and without using the frozen-index prior, LCB-SM consistently outperforms the Bernoulli, Bi-TpCM, and OPHM baselines in the tested configurations.

### 5.3. Effect of Receiver-Side FIP-OMP on the Reliability–Complexity Tradeoff

Based on the reduced inter-column correlation achieved by the proposed LCB-SM matrix, this subsection further evaluates the impact of the receiver-side FIP-OMP decoding algorithm in the LC-SVC architecture on the reliability and real-time performance of short-packet recovery. To isolate the effect of the decoder itself, the proposed LCB-SM spreading matrix is fixed in this subsection, and five sparse recovery algorithms, namely OMP, FIP-OMP, Dynamic OMP, MMP, and CoSaMP, are compared. The overall performance comparison between the conventional Bernoulli matrix and the proposed complete LC-SVC architecture will be presented in the subsequent joint performance analysis.

For a fair decoder-level comparison, all decoders process the same equivalent MRC output in each Monte Carlo block. Specifically, the transmitted sparse support, QPSK symbols, quasi-static Rayleigh fading coefficients, and AWGN realizations are kept identical for FIP-OMP, conventional OMP, Dynamic OMP, MMP, and CoSaMP. The receiver is equipped with $N_{r}=4$ antennas and employs perfect receiver-side CSI and normalized MRC. The horizontal axis is defined as the average pre-MRC SNR per receive branch.

The compared configurations are $\left(N,L_{s},K,b\right)=\left(16,12,2,6\right)$, (24,16,2,7), and (24,16,3,10). Here, *b* denotes the number of information bits mapped to one sparse vector. The MMP decoder uses a branch factor of 2 with at most 8 candidate paths, and the maximum number of iterations of CoSaMP is set to 10.

**Figure 7:**
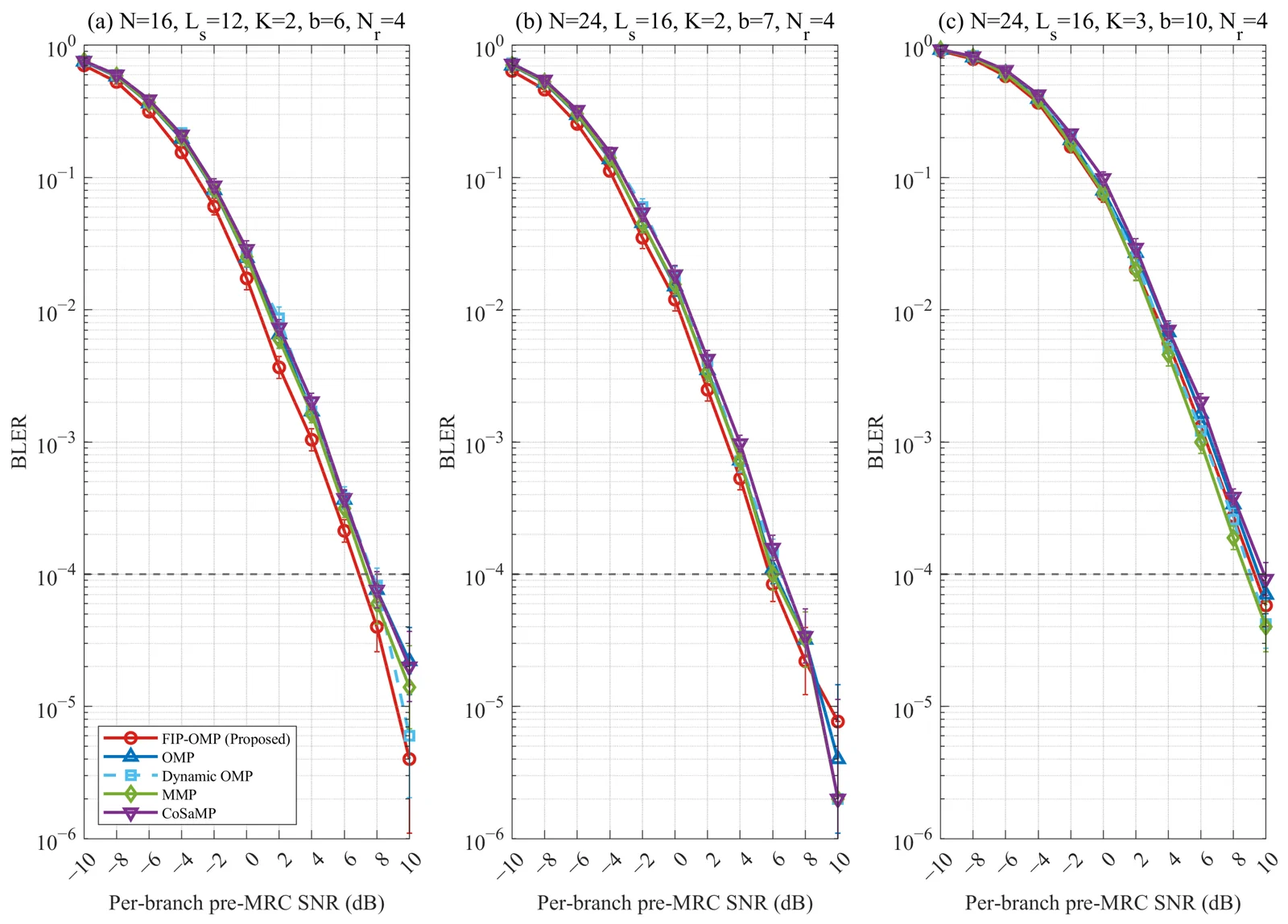
BLER performance comparison of five sparse recovery decoders with the proposed LCB-SM matrix under three system configurations.

Figure 7 shows the BLER performance of the five compared decoding algorithms under three representative sensing short-packet configurations. For the baseline configuration with N=16, $L_{s}=12$, and K=2, FIP-OMP achieves the lowest BLER over most of the tested SNR range. At the target BLER of $10^{-4}$, FIP-OMP reaches the threshold earlier than the other decoders, confirming the reliability gain introduced by the active/frozen-index pruning mechanism.

For the larger configuration $\left(N,L_{s},K,b\right)=\left(24,16,2,7\right)$, all decoders benefit from the low-coherence LCB-SM matrix, and their BLER curves are relatively close. FIP-OMP remains competitive over the low-to-medium SNR region and approaches the target BLER of $10^{-4}$ within the simulated SNR range. Dynamic OMP performs slightly better than conventional OMP in some SNR points, but it still does not outperform FIP-OMP overall.

The third configuration $\left(N,L_{s},K,b\right)=\left(24,16,3,10\right)$ further increases the sparsity level from K=2 to K=3. This setting is more challenging because the number of possible support combinations increases and the residual interference among selected atoms becomes stronger. As shown in Figure 7, all decoders exhibit higher BLERs than in the K=2 cases. MMP can obtain a slight BLER advantage at some high-SNR points due to its multi-path search mechanism. However, FIP-OMP remains close to the best-performing curves and still reaches the target BLER region within the simulated SNR range. Therefore, the proposed FIP-OMP maintains competitive reliability even when the sparse packet contains more active components.

It is worth noting that the performance improvement of FIP-OMP is not achieved by increasing the number of search paths. In contrast to MMP and CoSaMP, which rely on multi-path search or repeated support refinement, FIP-OMP exploits the frozen-index prior in the SVC mapping layer and prunes invalid indices before atom selection. This allows it to improve reliability without expanding the search tree.

The above BLER results show that FIP-OMP improves sparse support recovery by using the deterministic index structure of LC-SVC. Conventional OMP selects the atom with the largest correlation from all candidate columns, while FIP-OMP removes frozen indices from the candidate set in advance. This structural pruning reduces invalid searches and lowers the probability of false support detection.

**Figure 8:**
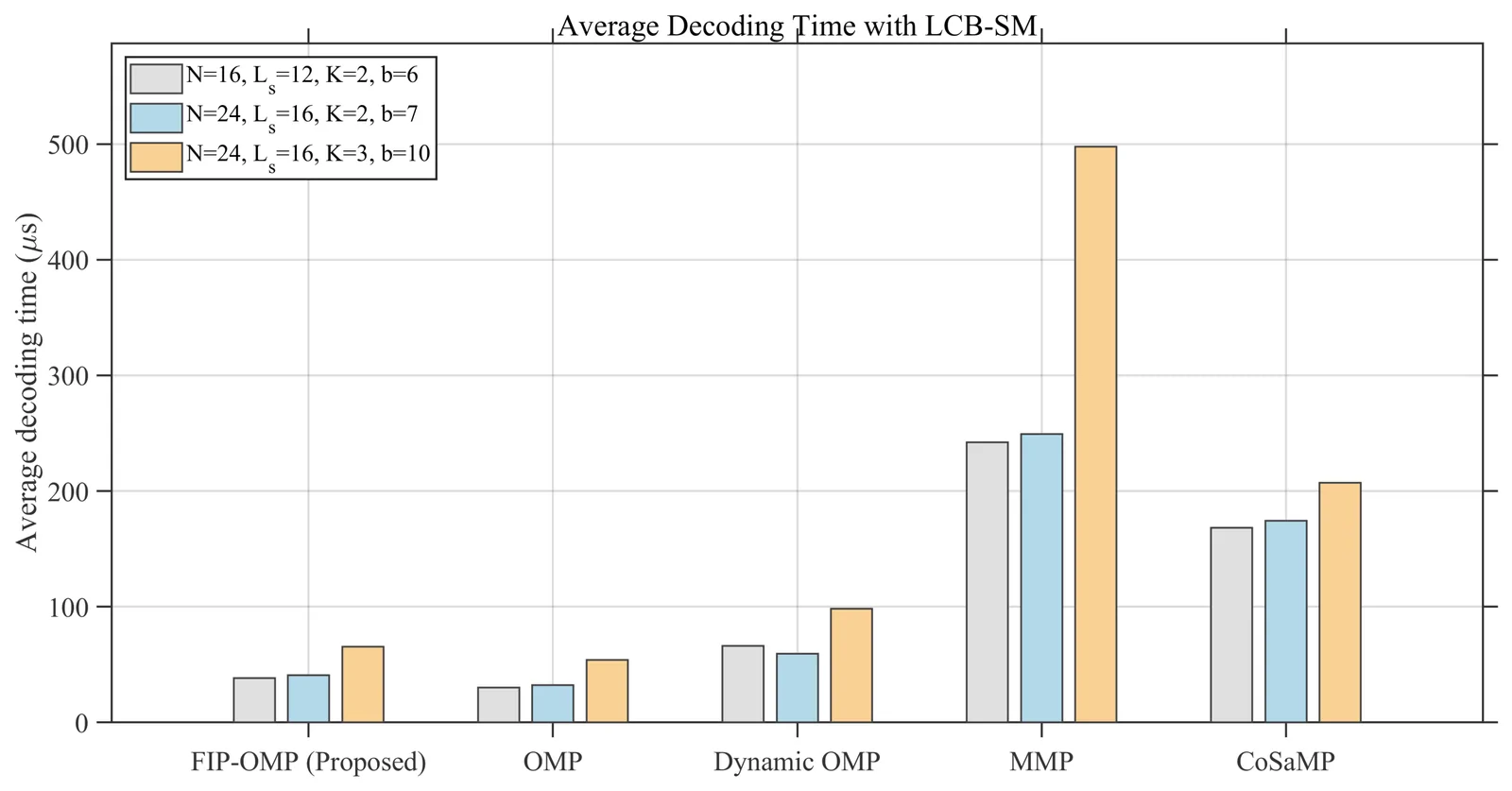
Average decoding time of five sparse recovery decoders with the proposed LCB-SM matrix under three system configurations.

In addition to recovery reliability, decoding latency is also a key metric in real-time wireless sensing systems. Figure 8 reports the average online decoding time of the compared decoders. Different from the accumulated running-time curve in the original evaluation, the revised figure directly reports the average decoding time per recovered block, which is more suitable for evaluating the real-time feasibility of short-packet sensing recovery. The decoding time is measured for the online sparse recovery stage after MRC combining, while the offline construction of LCB-SM is not included.

In addition to recovery reliability, decoding latency is also a key metric in real-time wireless sensing systems. Figure 8 reports the average online decoding time of the compared decoders. The decoding time is measured for the sparse recovery stage after MRC combining, and the offline construction of LCB-SM is not included.

The runtime difference mainly comes from the search strategy of each decoder. OMP and FIP-OMP use a greedy one-atom-at-a-time update, while Dynamic OMP increases the search burden by adapting the candidate set. In contrast, MMP maintains multiple candidate paths and CoSaMP repeatedly performs support expansion and pruning, which leads to higher computational cost.

It can be observed from Figure 8 that FIP-OMP remains close to conventional OMP in decoding time. For the three tested configurations, the average decoding time of FIP-OMP is approximately 38μs, 40μs, and 65μs, respectively, while Dynamic OMP is slower, with average decoding times of roughly 65μs, 59μs, and 98μs. MMP and CoSaMP are much slower than both OMP and FIP-OMP, especially for the K=3 case.

Overall, Figure 7 and Figure 8 jointly show that FIP-OMP provides a better reliability–complexity tradeoff than the other tested decoders. It improves BLER over OMP while keeping the decoding time close to OMP and clearly lower than Dynamic OMP, MMP, and CoSaMP.

### 5.4. Joint Performance and Component Ablation of LC-SVC

To further verify the overall gain achieved by jointly employing the transmitter-side LCB-SM matrix construction and the receiver-side FIP-OMP decoding algorithm, this subsection compares the complete LC-SVC architecture with the conventional Bernoulli+OMP SVC baseline.

Different from the matrix-level comparison in Section 5.2 and the decoder-level comparison in Section 5.3, this subsection evaluates the complete transmitter–receiver chain. The complete proposed scheme is denoted as LC-SVC, which combines LCB-SM at the transmitter and FIP-OMP at the receiver. The conventional SVC baseline uses a Bernoulli spreading matrix and conventional OMP decoding. All schemes are evaluated under the same MIMO-based short-packet transmission model with $N_{r}=4$ receive antennas, independent quasi-static Rayleigh fading, perfect receiver-side CSI, and normalized MRC. The horizontal axis is the average pre-MRC SNR per receive branch.

For a fair joint comparison, all schemes in the same Monte Carlo block use the same information state, sparse support mapping rule when applicable, QPSK symbols, channel realization, and noise realization. All binary spreading matrices are normalized by $1/\sqrt{K}$ so that the average transmitted chip power is the same. A block is declared correct only when the sparse support is exactly recovered. The reported BLER results are obtained from Monte Carlo simulations, and the statistical uncertainty is quantified by two-sided 95% Wilson confidence intervals.

**Figure 9:**
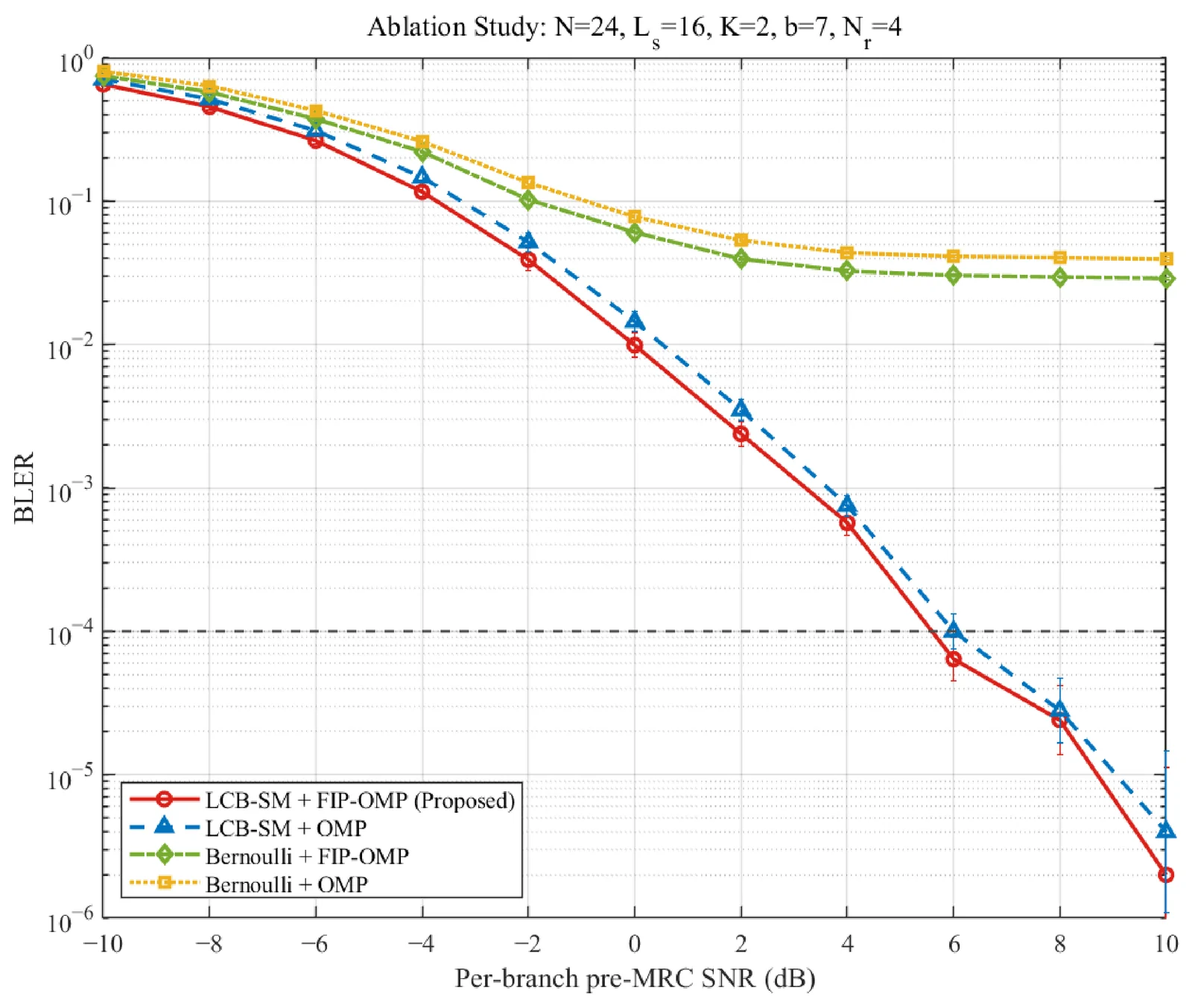
Joint BLER performance of the complete LC-SVC scheme and the conventional SVC baseline under different system configurations with $N_{r}=4$.

Figure 9 shows the BLER performance of the complete LC-SVC scheme and the conventional SVC baseline under different sparse-vector configurations.

The tested configurations are $\left(N,L_{s},K\right)=\left(16,12,2\right)$, (24,16,2), and (24,16,3), corresponding to different sparse-vector dimensions, spreading lengths, and sparsity levels. For each configuration, the complete LC-SVC scheme consistently achieves a much lower BLER than the conventional Bernoulli+OMP baseline.

For the configuration $\left(N,L_{s},K\right)=\left(16,12,2\right)$, the proposed LC-SVC curve decreases rapidly with SNR and reaches the target BLER region around $10^{-4}$ within the simulated range. In contrast, the Bernoulli+OMP baseline exhibits a clear high-SNR error floor and remains around $10^{-1}$. This confirms that the joint use of LCB-SM and FIP-OMP can effectively improve the reliability of short-packet support recovery under the baseline sparse-vector setting.

For the larger configuration $\left(N,L_{s},K\right)=\left(24,16,2\right)$, LC-SVC achieves even better high-SNR reliability. Around the target BLER of $10^{-4}$, the proposed scheme requires approximately 6 dB pre-MRC SNR, whereas the Bernoulli+OMP baseline does not reach this target within the simulated SNR range and remains at the level of several $10^{-2}$. This result verifies that LC-SVC remains effective when the sparse-vector dimension and the candidate support space are enlarged.

The configuration $\left(N,L_{s},K\right)=\left(24,16,3\right)$ further increases the sparsity level. Compared with the K=2 case, the recovery problem becomes more difficult because more active indices need to be jointly identified in each short packet. Nevertheless, the proposed scheme still reaches the target BLER region near the high-SNR end of the simulated range, whereas the Bernoulli+OMP baseline remains around $10^{-1}$. This demonstrates that the complete LC-SVC scheme is not limited to the K=2 case and can still provide a clear reliability advantage when more active sensing components are encoded in one sparse vector.

The joint comparison in Figure 9 also shows the effect of the system parameters on reliability. Increasing *N* enlarges the candidate index space and increases the number of possible support combinations, while increasing *K* makes sparse support detection more difficult. However, when the spreading length $L_{s}$ is properly chosen, the proposed low-coherence matrix construction and the frozen-index-pruned recovery algorithm can jointly maintain reliable support recovery. Therefore, the parameters $\left(N,L_{s},K,b\right)$ should be selected according to the desired payload size, compression ratio, and target BLER requirement.

To further clarify the individual contributions of the transmitter-side LCB-SM matrix and the receiver-side FIP-OMP decoder, a component ablation study is conducted under the representative configuration $\left(N,L_{s},K,b\right)=\left(24,16,2,7\right)$ with $N_{r}=4$.

**Figure 10:**
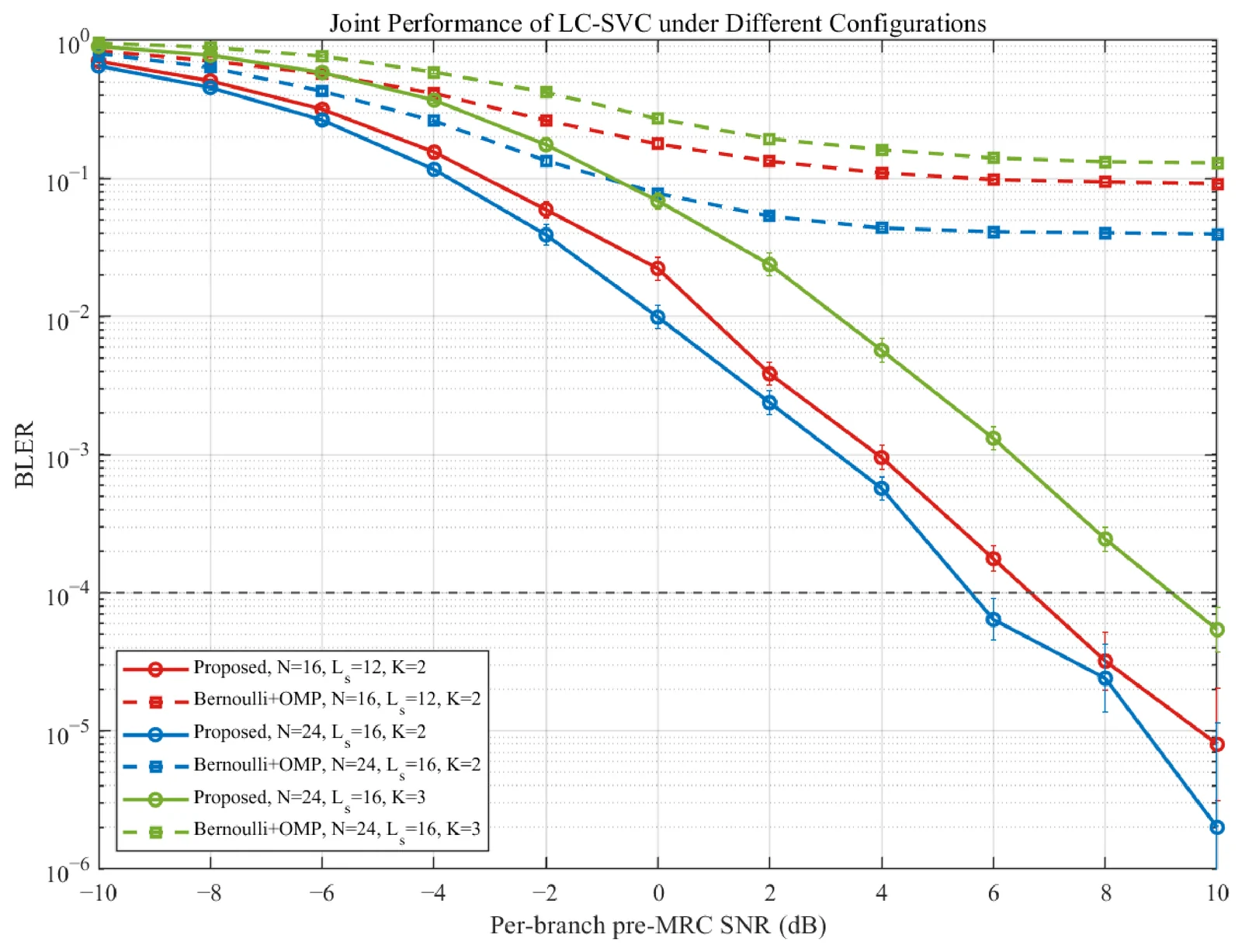
Joint performance of the proposed LC-SVC scheme and the conventional Bernoulli + OMP baseline under different configurations with $N_{r}=4$.

Figure 10 presents the component ablation study under the representative configuration $\left(N,L_{s},K,b\right)=\left(24,16,2,7\right)$. Four combinations are compared: LCB-SM with FIP-OMP, LCB-SM with conventional OMP, Bernoulli with FIP-OMP, and Bernoulli with conventional OMP. The first combination corresponds to the complete proposed LC-SVC scheme, whereas the last combination corresponds to the conventional SVC baseline. The other two combinations are used to separate the effects of the transmitter-side matrix construction and the receiver-side pruning decoder.

It can be observed that the two LCB-SM-based schemes significantly outperform the two Bernoulli-based schemes over the entire simulated SNR range. In particular, the Bernoulli-based schemes exhibit an evident high-SNR error floor around several $10^{-2}$, even when FIP-OMP is used at the receiver. In contrast, both LCB-SM-based schemes continue to decrease as the SNR increases and reach the BLER region below $10^{-4}$. This result indicates that the low-coherence spreading matrix is the dominant factor in suppressing high-SNR support ambiguity. If the spreading matrix contains highly correlated columns, receiver-side pruning alone cannot completely remove the error floor caused by matrix-induced support confusion.

The comparison between LCB-SM with FIP-OMP and LCB-SM with OMP further shows the additional contribution of the proposed receiver-side decoder. Around the target BLER of $10^{-2}$, the complete LC-SVC scheme reaches the target at approximately 0 dB, while LCB-SM with conventional OMP requires a slightly higher SNR. In the medium-to-high SNR region, FIP-OMP provides a consistent improvement over conventional OMP by excluding frozen indices from the atom selection set. Although the gain is smaller than that introduced by replacing the Bernoulli matrix with LCB-SM, it is achieved with only a small increase in online decoding complexity, as shown in the decoder-level comparison in the previous subsection.

These results demonstrate that LCB-SM and FIP-OMP exhibit good complementarity. LCB-SM reduces the inter-column correlation of the spreading matrix at the transmitter, thereby improving the physical-layer distinguishability of different sensing-state indices. FIP-OMP exploits the frozen-index prior at the receiver to suppress invalid support searches and reduce noise-induced incorrect atom selection. When combined, these two components effectively alleviate the reliability bottleneck in resource-constrained sensing short-packet transmission.

The ablation result further confirms this complementarity. LCB-SM improves the geometric distinguishability among sparse support indices by reducing inter-column correlation at the transmitter side, while FIP-OMP improves the receiver-side search process by avoiding invalid candidate indices imposed by the deterministic sparse mapping rule. Therefore, the complete LC-SVC scheme benefits from both a more distinguishable binary spreading matrix and a more constrained support search space.

Overall, the ablation and joint-performance results verify the effectiveness of the complete LC-SVC architecture. The transmitter-side LCB-SM mainly reduces matrix-induced support ambiguity, while the receiver-side FIP-OMP further reduces redundant searches over invalid indices. Their combination enables the proposed LC-SVC scheme to achieve substantially lower BLER than the conventional Bernoulli+OMP SVC baseline under multiple short-packet configurations. These results demonstrate that LC-SVC provides a reliable and low-complexity solution for MIMO-based wireless sensor systems with short-packet transmission requirements.

### 5.5. SDR-Based Wireless Reconstruction Experiment for Force-Feedback Sensing Data

To verify the feasibility of the proposed LC-SVC scheme in a real wireless sensing scenario, an experimental platform is established to evaluate the transmission and reconstruction performance of force-feedback sensing data [7,8,10]. This experiment targets typical short-packet wireless sensing applications, such as tactile feedback, force feedback, and industrial interaction control, where continuous force-feedback sensing data are uploaded to an edge processing unit through a wireless link [7,8,9]. Different from simulation-based evaluations with randomly generated data, force-feedback data are continuous physical measurements. Transmission errors may not only lead to the failure of individual data block recovery, but also introduce abnormal fluctuations in the time-domain sequence, thereby affecting the stability of tactile feedback. Therefore, a real sensing-data reconstruction experiment can more directly validate the engineering applicability of the proposed scheme in wireless sensing systems.

**Figure 11:**
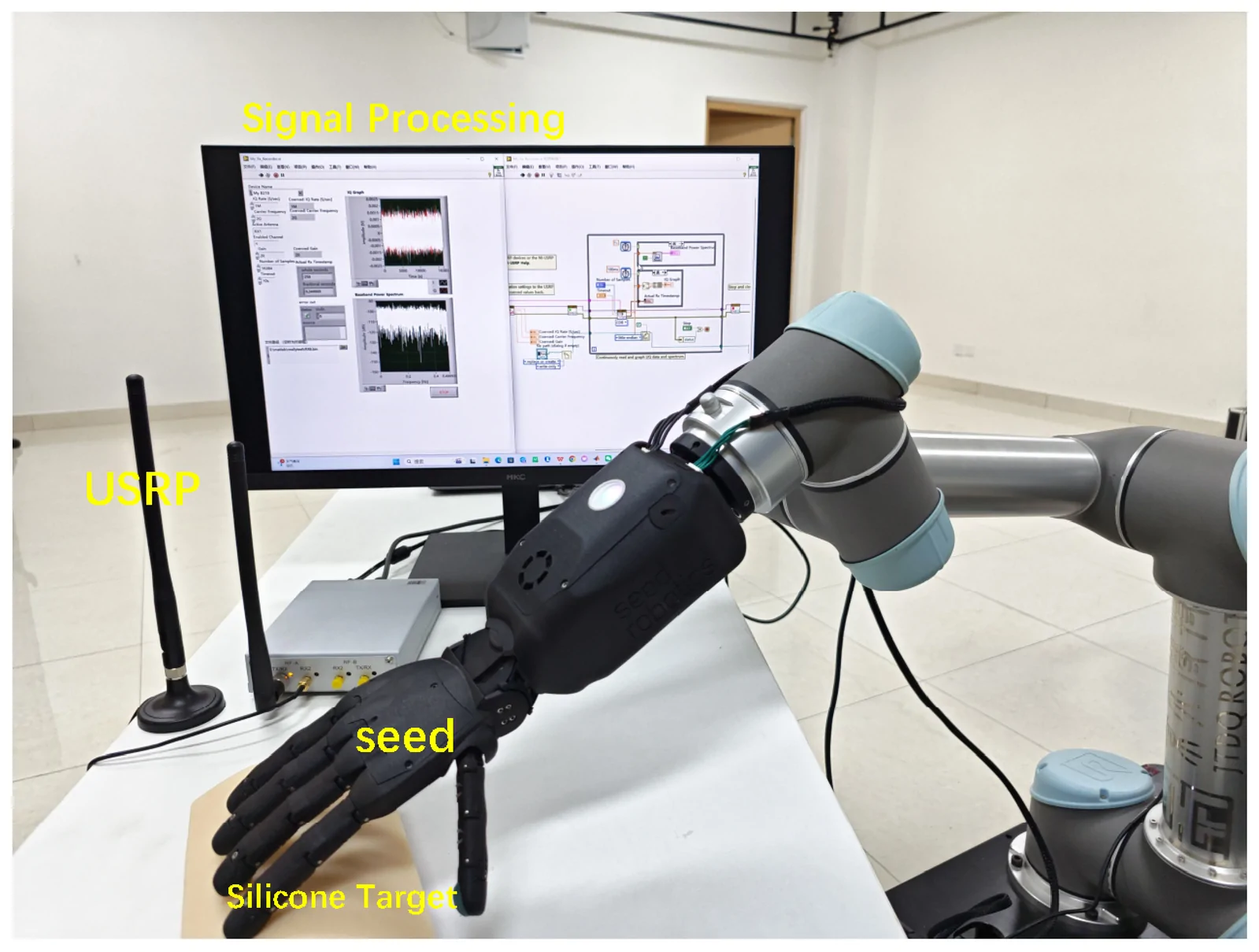
Schematic diagram of the SDR-based experimental platform.

The experimental platform is shown in Figure 11, and consists of a tactile signal acquisition terminal, an SDR wireless link, and a signal processing terminal. The tactile acquisition device is the SEED Robotics RH8D dexterous hand, which has 12 active degrees of freedom and integrates pressure sensors at the fingertips. The force resolution is 0.1 N, and high-speed USB/RS485 communication interfaces are supported. The wireless transmission link is implemented based on the USRP-LW B210 software-defined radio peripheral. This platform can be used to emulate the equivalent short-packet receiving link in a MIMO-based wireless sensing gateway and to verify the robustness of the transmitter-side encoding module and receiver-side decoding module in the proposed architecture over real physical channels.

In the experiment, the range of the collected raw force-feedback data is 0–3441 mN. To adapt to short-packet transmission and improve index utilization, the raw force data are first linearly mapped to the effective numerical range of 0–35, and then decomposed into the integer part and two decimal components for parallel transmission. The proposed SVC scheme maps three groups of 6-bit information into a joint sparse vector with N=16 and K=2, forming a 48-dimensional joint sparse representation, which is finally projected into a 32-bit observation space. At the receiver, the FIP-OMP algorithm is adopted to recover the sparse support set, and the reconstructed force-feedback sensing sequence is obtained through inverse index mapping.

**Figure 12:**
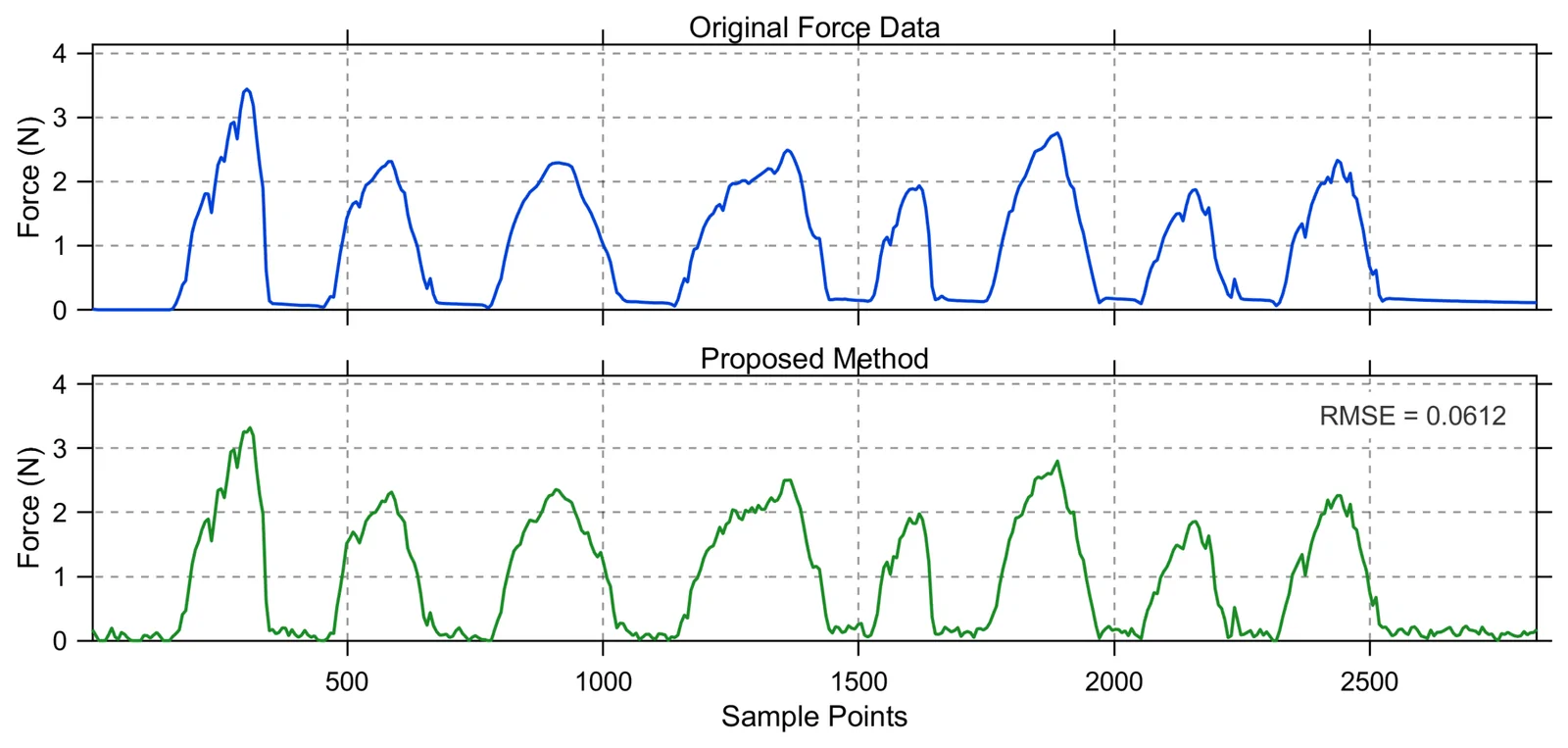
Real force-feedback sensing data and wireless reconstruction results.

Figure 12 presents the real force-feedback sensing data and the corresponding reconstruction results after wireless transmission. It can be observed that the proposed scheme accurately follows the main variation trend of the original force-feedback curve and maintains high consistency during multiple contact peaks and release phases. Since FIP-OMP freezes the invalid index set during the decoding stage, noise-induced invalid state decisions are effectively suppressed. As a result, the reconstructed sequence preserves good temporal continuity overall. This property is particularly important for applications such as tactile feedback and robotic teleoperation, where abrupt fluctuations in force-feedback data may lead to discontinuous operator perception or unstable control inputs [7,8].

To further quantitatively evaluate the reconstruction performance from the perspective of sensor data quality, the RMSE is adopted to measure the deviation between the reconstructed force-feedback sequence and the original sensing sequence:

(32) $$\mathrm{RMSE}=\sqrt{\frac{1}{T}\sum_{t=1}^{T}{\left(f_{t}-{\hat{f}}_{t}\right)}^{2}},$$

where $f_{t}$ denotes the *t*-th original force-feedback sensing value, ${\hat{f}}_{t}$ denotes the corresponding reconstructed value after wireless transmission, and *T* is the number of sampling points. The experimental results show that the reconstruction error of the proposed LC-SVC scheme is ${\mathrm{RMSE}}_{\mathrm{svc}}=0.0612$. This result indicates that, under a real SDR-based wireless link, the proposed scheme can recover continuous force-feedback sensing data with a low reconstruction error.

The above experimental results demonstrate that the proposed scheme not only reduces the BLER of sensing short packets in simulations, but also enables stable reconstruction of force-feedback sensing data over a real wireless channel. Since LCB-SM employs an offline-optimized binary spreading matrix and FIP-OMP only introduces a lightweight frozen-index masking operation, the proposed scheme is suitable for deployment in low-power sensing nodes and edge wireless gateways. Therefore, it can provide practical engineering support for real-time short-packet transmission in tactile Internet, robotic teleoperation, and MIMO-based wireless sensing systems.

## 6. Conclusions

This paper proposed LC-SVC, a low-complexity sparse vector coding scheme for reliable short-packet transmission in MIMO-based wireless sensing systems. LC-SVC combines LCB-SM, which constructs a low-coherence binary spreading matrix through Hadamard initialization and column-wise correlation optimization, with FIP-OMP, which exploits frozen indices to suppress invalid atom selection during sparse recovery. Simulation results showed that LCB-SM and FIP-OMP improve BLER performance while maintaining low encoding and decoding complexity. An SDR-based testbed using a USRP-LW B210 and a SEED Robotics RH8D dexterous hand further verified the reconstruction of continuous fingertip force-sensing data with an RMSE of 0.0612. These results demonstrate that LC-SVC can effectively improve the reliability of MIMO-based short-packet recovery under the considered configurations, while keeping the transmitter and receiver complexity low. The present evaluation assumes perfect receiver-side CSI and does not include pilot overhead. Future work will investigate LC-SVC under imperfect CSI and more practical sensing scenarios.

## Figures and Tables

**Table 1:** Principal simulation parameters.

Parameter	Setting
Channel model	Independent quasi-static Rayleigh fading with complex AWGN
Receive antennas	$N_{r}=4$
CSI and combining	Perfect receiver-side CSI and normalized MRC
SNR definition	Average pre-MRC SNR per receive branch
SNR range	−10 to 10 dB, with a 2 dB interval for matrix comparison
Sparse coefficients	Independent unit-energy QPSK symbols
Matrix normalization	$C=C_{\mathrm{bin}}/\sqrt{K}$
Compared matrices	LCB-SM, Bernoulli, Bi-TpCM, and OPHM
Matrix-level decoder	Conventional OMP
Decoder-level decoders	FIP-OMP, OMP, MMP, and CoSaMP
Matrix-level configs	(16,12,2), (24,12,2), (24,16,2), (32,16,2)
Sensitivity cases	(24,16,3) and (32,16,3)
Decoder-level configs	(16,12,2,6), (24,16,2,7), (24,16,3,10)
LCB-SM construction	t=100; initial threshold 0; increment 1; implicit enumeration
Random realizations	10 for Bernoulli and OPHM
Statistical reporting	BLER and 95% Wilson intervals
Decoder parameters	MMP branch factor 2 with at most 8 paths; CoSaMP maximum iterations 10

## Data Availability

The simulation data and experimental data supporting the findings of this study are available from the corresponding author upon reasonable request.
